# Urban mining by flash Joule heating

**DOI:** 10.1038/s41467-021-26038-9

**Published:** 2021-10-04

**Authors:** Bing Deng, Duy Xuan Luong, Zhe Wang, Carter Kittrell, Emily A. McHugh, James M. Tour

**Affiliations:** 1 Department of Chemistry, Rice University, Houston, TX 77005 USA; 2 Smalley-Curl Institute, Rice University, Houston, TX 77005 USA; 3 NanoCarbon Center and the Welch Institute for Advanced Materials, Rice University, Houston, TX 77005 USA; 4 Department of Materials Science and NanoEngineering, Rice University, Houston, TX 77005 USA

**Keywords:** Pollution remediation, Batteries

## Abstract

Precious metal recovery from electronic waste, termed urban mining, is important for a circular economy. Present methods for urban mining, mainly smelting and leaching, suffer from lengthy purification processes and negative environmental impacts. Here, a solvent-free and sustainable process by flash Joule heating is disclosed to recover precious metals and remove hazardous heavy metals in electronic waste within one second. The sample temperature ramps to ~3400 K in milliseconds by the ultrafast electrical thermal process. Such a high temperature enables the evaporative separation of precious metals from the supporting matrices, with the recovery yields >80% for Rh, Pd, Ag, and >60% for Au. The heavy metals in electronic waste, some of which are highly toxic including Cr, As, Cd, Hg, and Pb, are also removed, leaving a final waste with minimal metal content, acceptable even for agriculture soil levels. Urban mining by flash Joule heating would be 80× to 500× less energy consumptive than using traditional smelting furnaces for metal-component recovery and more environmentally friendly.

## Abstract (web summary)

Precious metals recovery from electronics wastes, termed urban mining, is significant for a circular economy. Here, the authors reported a solvent-free and ultrafast process based on flash Joule heating to recover precious metals and remove toxic heavy metals in electronic wastes.

## Introduction

More than 40 million tons of electronic waste (e-waste) are produced globally each year^1,2^, which is the fastest-growing component of solid wastes due to the rapid upgrade of personal electrical and electronic equipments^3,4^. Most e-waste is landfilled with only ~20% being recycled^5^, which could lead to negative environmental impact due to the broad use of heavy metals in electronics^6–8^. E-waste could become a sustainable resource because it contains abundant valuable metals^9^. The concentrations of some precious metals in e-waste are higher than those in ores^1^. Precious metals recovery from e-waste, termed urban mining, is becoming more cost-effective than virgin mining^2^ and important for a circular economy^8^. Similarly, due to the broad use of heavy metals in electronics, including Cd, Co, Cu, Ni, Pb, and Zn, e-waste could lead to significant health risks and negative environmental impact^6–8^. The heavy metal leakage due to improper landfill disposal leads to environmental disruption^1,8^. The release of hazardous components during the recycling processes in the form of dust or smoke^6^ deteriorates the health of recycling workers and local residents. For example, a significantly higher concentration of Pb has been found in the blood of e-waste workers^7,10^.

The lack of high-yielding and environmentally friendly recovery processes are the main obstacles to urban mining^9^. The traditional method for e-waste recycling is based on a pyrometallurgy process^11^, where metals are melted by heating at high temperature. Pyrometallurgy is energy-intensive, lacks selectivity, and requires high-grade precursors^12^. Pyrometallurgical processes also produce hazardous fumes containing heavy metals, especially for those with low melting points such as Hg, Cd, and Pb^9^. The hydrometallurgical process is more selective and done by leaching the metals using acid, base, or cyanide^13^. The leaching kinetics are usually slow. The use of highly concentrated leaching agents renders the hydrometallurgical process difficult for large-scale applications, and large amounts of liquid waste and sludge are produced that could result in secondary pollution^14^. Biometallurgy could be highly selective and environmentally sustainable, yet it is still in its infancy^15^. The separation of valuable metals from various materials matrices, including plastics, glass, and ceramics, are based upon their differences in physical or chemical properties. For example, the gravity separation technique relies on differing specific densities^16^. Magnetic separation is used to separate magnetic metals from nonferrous waste^17^. Hydrometallurgical separation is based upon the chemical reactivity of metals with leaching agents^18^.

Here, we show that the different vapor pressure of metals compared to that of substrate materials (carbon, ceramics, and glass) enables the separation of metals from e-waste. This is termed evaporative separation. The high vapor pressure of precious metals is obtained by an ultrafast flash Joule heating (FJH) process under vacuum. A subsecond current pulse is passed through the precursors, which brings the sample to an ultrahigh temperature of ~3400 K, enabling the evaporative separation of precious metals. Halide additives are used to improve the recovery yield to >80% for Rh, Pd, and Ag, and >60% for Au that are abundant in the tested e-waste. Alternatively, compared with directly leaching e-waste raw materials, by leaching the residual solids after FJH, the recovery yield is significantly improved with tens of times increase for Ag and few times increase for Rh, Pd, and Au. The toxic heavy metals, including Cd, Hg, As, Pb, and Cr, could also be removed and collected, minimizing the health risks and environmental impact of the recycling process.

## Results

### Evaporative separation of precious metals from e-waste by FJH

**Fig. 1:**
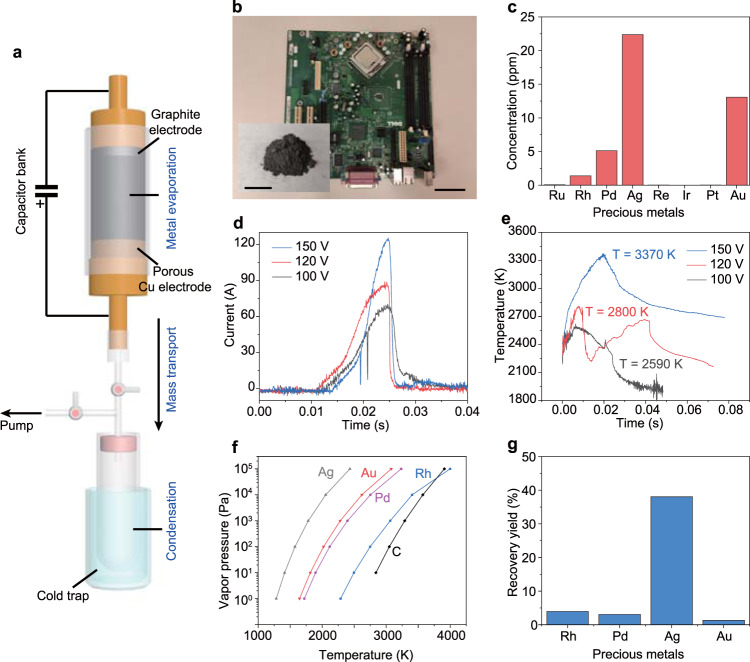
Recovery of precious metals by flash Joule heating (FJH). **a** Schematic of the FJH and evaporative separation system. The system was composed of three parts, including FJH for metal evaporation, the vacuum system for mass transport, and the cold trap for volatiles condensation. **b** Picture of a printed circuit board (PCB). Scale bar, 5 cm. Inset, the mixture of carbon black (CB) with PCB powder. Scale bar, 2 cm. **c** Concentrations of precious metals in PCB as determined by inductively coupled plasma mass spectrometry (ICP-MS). **d** Currents vs time recorded under different FJH voltages. **e** Real-time temperature measurements at different FJH voltages by fitting blackbody radiation emitted from the sample. **f** Vapor pressure–temperature relationship of precious metals and carbon. **g** Recovery yield of precious metals by condensing the evaporated gas components. The recovery yield is the average of three independent FJH experiments (*n* = 3).

The FJH process to recover precious metals from e-waste involves three stages (Fig. 1a). The metals in e-waste were heated and evaporated by ultrahigh-temperature FJH, then the metal vapors were transported under vacuum and collected by condensation. A printed circuit board (PCB) from a discarded computer, a representative e-waste, was used as the starting material (Fig. 1b and Supplementary Fig. 1). The PCB was ground to small powder and mixed with carbon black (CB), which served as the conductive additive (Fig. 1b, inset). To establish baseline concentrations, the PCB was digested using dilute aqua regia^19^, and the concentration of precious metals was determined by inductively coupled plasma mass spectrometry (ICP-MS). Among the precious metals, Rh, Pd, Ag, and Au are abundant with concentrations of several to tens of parts per million (ppm) (Fig. 1c).

In a typical FJH process, the mixture of PCB powder and ~30 wt% CB was slightly compressed inside a quartz tube between two sealed electrodes (Fig. 1a and Supplementary Fig. 2). One electrode was a porous Cu electrode to facilitate gas diffusion, and the other was a graphite rod (Supplementary Fig. 3). The resistance of the sample was tunable by adjusting the compressive force on the two electrodes. The two electrodes were connected to a capacitor bank with a total capacitance of 60 mF (Supplementary Fig. 3). The detailed separation conditions are shown in Supplementary Table 1. The high-voltage discharge of the capacitor bank brings the reactant to a high temperature. With the fixed sample resistance of ~1 Ω, the current passing through the sample was measured under different FJH voltages (Fig. 1d). The real-time temperature of the sample was estimated by fitting the blackbody radiation in the 600–1100 nm emission (Supplementary Fig. 4). The temperature varied according to the FJH voltage, reaching ~3400 K at 150 V in <50 ms (Fig. 1e). Since the resistance of the sample is much larger than that of the graphite and porous Cu electrode, the voltage drop was mainly imposed on the sample. Hence, the high-temperature region was limited to the sample and the FJH setup has good durability even though it can achieve a high temperature of >3000 K (Supplementary Fig. 5). Numerical simulations showed that the temperature was relatively uniform along both the longitudinal and radial directions of the sample (Supplementary Note 1, temperature simulation, Supplementary Fig. 6), demonstrating the homogenous heating ability of the FJH process.

Such a high temperature (>3000 K) volatilizes most of the non-carbon components. According to the calculated vapor pressure–temperature relationships (Fig. 1f), the precious metals have a higher vapor pressure than carbon, the latter not subliming until ~3900 K^20^. As a result, the metals are evaporated, and the major carbon-containing components such as plastics were carbonized^21,22^. The evaporated metal vapors were captured by condensation in a cold trap (Fig. 1a and Supplementary Fig. 2). Some of the vapor remained gaseous even at the liquid N_2_ temperature (77 K) (Supplementary Fig. 2); these gases were presumed to be H_2_ and CO^22^. The content of the precious metals in the condensed solid was measured and the recovery yield was calculated (Fig. 1g and Supplementary Note 2). The recovery yield of Ag was ~40%, while Rh, Pd, and Au had a relatively low recovery yield of ~3%. This is because Ag has a high vapor pressure and relatively low boiling point (Supplementary Fig. 7). The concentration of precious metals in the starting commercial CB is 1–2% of the concentration in PCB, hence their presence in CB will not introduce significant errors (Supplementary Fig. 8). Moreover, the precious metals tend to not form stable carbide phases even at high temperature due to their extremely low C solubility^23^ (Supplementary Fig. 9). Hence, the use of CB as a conductive additive will not affect the evaporative behavior of precious metals.

### Halide assisted improvement of recovery yield

**Fig. 2:**
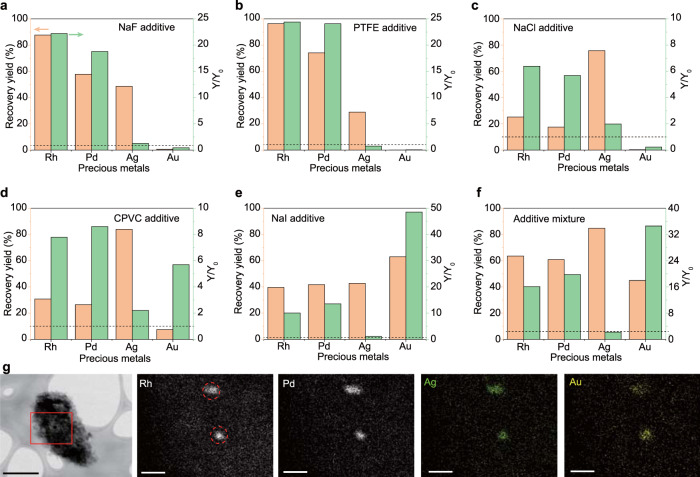
Halide assisted improvement of recovery yield. Recovery yield of precious metals by using (**a**) NaF, (**b**) PTFE, (**c**) NaCl, (**d**) CPVC, (**e**) NaI, and (**f**) mixture of NaF, NaCl, and NaI, as additives. *Y*_0_ and *Y* mean the recovery yield of precious metals without and with additives, respectively. The dashed line denotes *Y*/*Y*_0_ = 1, meaning that there is no advantage of the additive if *Y*/*Y*_0_ $\le\,$1. The recovery yields were the average of three independent flash Joule heating (FJH) experiments (*n* = 3). **g** Scanning transmission electron microscopy (STEM) image of the collected solids, and energy dispersive spectroscopy (EDS) maps of Rh, Pd, Ag, and Au at the rectangular region. Scale bar in STEM image, 0.5 μm; scale bars in EDS maps, 100 nm. The dashed circles in Rh show the clustered alloys.

The high-recovery yield of the evaporative separation relies on the generation of more volatile components. To improve the recovery, halides were used as additives because of the much higher vapor pressure of metal halides compared with the elemental metals (Supplementary Fig. 10)^24^. Fluorine-containing components were first used as the additive, including sodium fluoride (NaF) and polytetrafluoroethylene (PTFE, Teflon). With the additives, the recovery yields of Rh and Pd were improved to >80% and 70%, respectively (Fig. 2a, b and Supplementary Note 2), demonstrating ~20× improvement compared to the experiments without additives. The concentration of precious metals in the additives was <2% of those in PCB (Supplementary Fig. 11), hence we can exclude the additives from introducing significant error in the recovery of precious metals. Chlorine-containing compounds were tried because of their abundance and low cost. Both sodium chloride (NaCl) and potassium chloride (KCl) were used (Fig. 2c and Supplementary Fig. 12). The recovery yields of Rh, Pd, and Ag increased for both NaCl and KCl additives. In addition, both polyvinyl chloride (PVC) and chlorinated polyvinyl chloride (CPVC) plastics were used (Fig. 2d and Supplementary Fig. 12). The recovery yield of all four precious metals was increased, especially for Ag, with the recovery yield improving to >80%. The plastic additives were ground post-consumer samples with very low or negative values, so they will not introduce significant materials cost during the e-waste recycling process.

Even with the F and Cl additives, the recovery yield of Au is <10%. Interestingly, the recovery yields of all four precious metals were improved when sodium iodide (NaI) was used as the additive; the recovery yield of Au was improved to >60% (Fig. 2e). The I additive has the best performance among halides for Au recovery. According to the hard and soft acids and bases (HSAB) theory, Au^+^ is a soft Lewis acid, and I^−^ is a soft Lewis base while F^−^ and Cl^−^ are harder than I^−^ ^25^, favoring AuI. By using an additive mixture of NaF, NaCl, and NaI, the precious metals all had a good recovery yield, >60% for Rh, >60% for Pd, >80% for Ag, and >40% for Au (Fig. 2f). The composition analysis of the raw materials and the remaining solid after FJH by X-ray photoemission spectroscopy (XPS) showed that 10–40% of the halide additives were evaporated during the FJH process (Supplementary Fig. 13), which could be recovered and reused by a water washing and precipitation process.

We conducted a total composition analysis of the collected metals in the cold trap (Supplementary Note 3). In both cases with or without the chemical additives, in addition to the precious metals, the most abundant metals were Cu with mass ratio >60 wt%, followed by other prominent metals in e-waste including Al, Sn, Fe, and Zn (Supplementary Fig. 14). Further purification and refining could be done by selective precipitation, solvent extraction, and solid-phase extraction, which are commercially well-established practices^26^.

The morphology and chemical composition of the condensed solids were characterized using scanning transmission electron microscopy (STEM) and energy dispersion spectroscopy (EDS). The elemental maps showed the clustered alloy particles of Rh, Pd, Ag, and Au (Fig. 2g), which were formed by the ultrafast heating and rapid cooling of the FJH process. This is similar to the case of the carbothermic shock synthesis of high-entropy alloy nanoparticles, which could be potentially used in catalysts^27^. In other regions, the precious metals spreading over the entire product were also observed (Supplementary Fig. 15). Moreover, the XPS analysis of the collected volatiles showed that Ag and Au were mainly in the elemental state, while elemental state and higher oxidation state coexisted for Rh and Pd, presumably due to their different chemical reactivity (Supplementary Note 3 and Supplementary Fig. 16).

### Improved leaching efficiency of precious metals by FJH

**Fig. 3:**
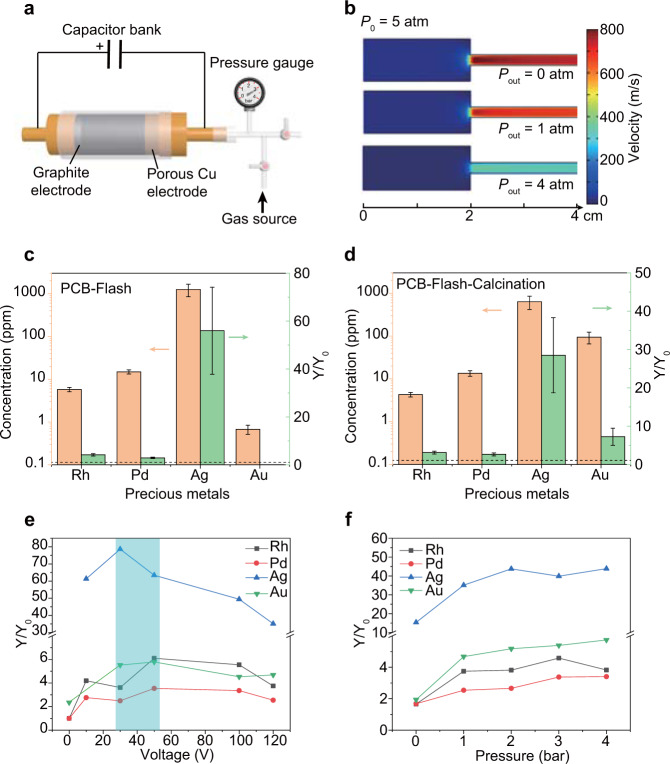
Leaching efficiency improvement of precious metals by the flash Joule heating (FJH) process. **a** Schematic of the pressurized setup for FJH. **b** Gas flow simulation under different pressures. The inner pressure (*P*_0_) during the FJH was calculated to be ~5 atm. *P*_out_ of 0 atm, 1 atm, and 4 atm correspond to the FJH under vacuum, atmospheric pressure, and 3 atm of positive pressure. **c** Concentration of precious metals and improvement of recovery yield by FJH. *Y*_0_ and *Y* mean the recovery yield by leaching printed circuit board (PCB) and PCB-Flash, respectively. The dashed line denotes *Y/Y*_0_ = 1. The error bars denote the standard deviation where *n* = 3. **d** Concentration of precious metals and improvement of recovery yield by FJH and calcination. *Y*_0_ and *Y* mean the recovery yield by leaching PCB and PCB-Flash-Calcination, respectively. The dashed line denotes *Y/Y*_0_ = 1. The error bars denote the standard deviation where *n* = 3. **e** Improvement of recovery yield varied with FJH voltages under atmospheric pressure. The highlighted region is the approximate optimal voltage for all metal recovery. **f** Improvement of recovery yield varied with pressure. For **e** and **f**, the recovery yields of Rh, Pd, and Ag are calculated from PCB-Flash, and the recovery yield of Au is calculated from PCB-Flash-Calcination.

Apart from the condensation of the volatile composition, the other pathway to recover the precious metals was by leaching the residual solids obtained by FJH (Supplementary Fig. 17a). Different from the use of a vacuum to facilitate the metal volatilization in the evaporative separation scheme (Fig. 1a), a pressurized setup was built to trap the metals in the reactor (Fig. 3a). An inert gas (N_2_) cylinder was connected to the FJH reactor, where the pressure was monitored by a pressure gauge. The inner pressure (*P*_0_) during FJH was estimated to be ~5 atm according to the amount of collected gas (Supplementary Fig. 2 and Supplementary Note 1). Based on the pressure drop and the size of the FJH chamber, the gas diffusion was simulated under different pressures (*P*_out_) (Fig. 3b, Supplementary Fig. 18). When vacuum was used (*P*_out_ = 0 atm), as it is in the evaporative separation (Fig. 1a), the gas velocity was up to 800 m s^−1^. Such a high gas velocity aided the volatile components to quickly diffuse to the cold trap and prevent the condensation loss at the tube sidewalls. In contrast, the gas velocity was greatly reduced with the increase in pressure (Fig. 3b). As a result, more of the originally volatile components were trapped within the residual solids in the reactor. The detailed reaction conditions for the pressurized FJH are shown in Supplementary Table 2.

We started from leaching the residual solids after FJH (denoted as PCB-Flash) at 120 V and atmospheric pressure using dilute acids (1 M HCl, 1 M HNO_3_) (Supplementary Fig. 17a). The leachable content of Rh, Pd, and Ag in PCB-Flash was substantially higher than that in the PCB raw materials (Fig. 3c). The ratio of the recovery yield by leaching the PCB-Flash (*Y*) and leaching the PCB raw materials (*Y*_0_) was calculated. FJH with leaching was far more effective than leaching alone. The recovery yield of Rh, Pd, and Ag was increased by 4.17 ± 0.48, 2.90 ± 0.31, 56.0 ± 18.1 times, respectively (Fig. 3c). The deviations could be from the inhomogeneous distribution of precious metals in e-waste. Interestingly, the Au recovery yield was reduced after the FJH process. The reason was presumably the formation of covalent bonds between Au and carbon^28^, which could significantly increase the difficulty of acid leaching. The thermogravimetric analysis (TGA) of the PCB-Flash showed that the carbon could be removed in the air at ~700 °C (Supplementary Fig. 17b). Hence, the PCB-Flash solid was calcined at 700 °C for 1 h (denoted as PCB-Flash-Calcination, Supplementary Fig. 17b). The PCB raw materials were also calcined as a control (denoted as PCB-Calcination, Supplementary Fig. 17c). The XPS analysis showed the efficient removal of carbon by calcination (Supplementary Fig. 17d). With the FJH and calcination process, the recovery yields of Rh, Pd, Ag, and Au were increased by 3.11 ± 0.37, 2.64 ± 0.39, 28.5 ± 9.8, 7.24 ± 2.22 times, respectively (Fig. 3d). The values are larger than those achieved with the calcination-only process (Supplementary Figs. 17e, f).

The presumable mechanism of the improved leaching efficiency by FJH is shown in Supplementary Fig. 19. Modern electronics are fabricated and packaged by a planar process and have a laminated configuration, where the useful metals are embedded into the polymer or ceramic matrices (Supplementary Fig. 19a)^13^. Even after the pulverization, the particle size was large ~5 μm (Supplementary Fig. 19b). The laminated structure hinders the extraction of metals in a typical hydrochemical process, resulting in elongated leaching times and low leaching efficiencies^13^. During the FJH process, the matrix was rendered as an ultrafine powder at the ultrahigh temperature (Supplementary Figs. 19c, d), and the metals were exposed (Supplementary Fig. 19e), which greatly accelerated the leaching rate and extent of metal extraction.

The effects of the FJH voltage and pressure on the recovery yield were studied. It was found that the modest FJH voltages between 30 and 50 V led to the best recovery yield (Fig. 3e). Too low voltage did not provide enough energy to thermally decompose the matrix, while too high voltage presumably resulted in the evaporative loss. It was found that a higher surrounding pressure was beneficial (Fig. 3f). This is because the volatile components were trapped in the residual solid, as we projected by the gas- flow simulations (Fig. 3b). The mild acid-leaching condition (1 M HCl, 1 M HNO_3_) used in our process is more cost-effective and environmentally friendly compared to other hydrometallurgical processes, which use highly concentrated mineral acids such as aqua regia^13,29^, or toxic cyanides^18,30^ as extractants for achieving a high-recovery yield.

### Removal and collection of toxic heavy metals in e-waste

**Fig. 4:**
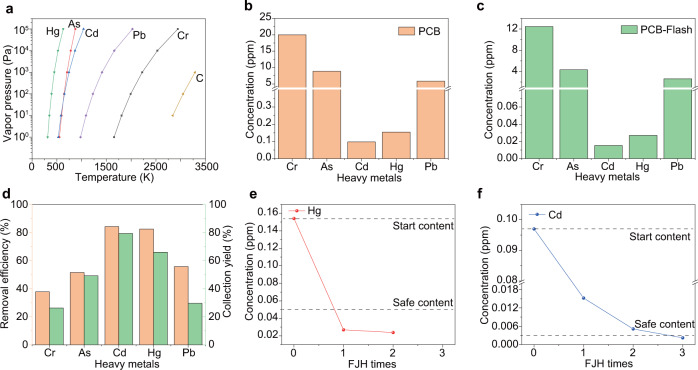
Removal of heavy metals in e-waste by flash Joule heating (FJH) process. **a** Vapor pressure–temperature relationships of toxic heavy metals and carbon. **b** Concentrations of toxic heavy metals in the printed circuit board (PCB). **c** Concentrations of toxic heavy metals in PCB after FJH. **d** Removal efficiency and collection yield of heavy metals. The efficiency and yield were the average of three independent FJH experiments (*n* = 3). **e** Concentration of Hg in the residues after multiple FJH reactions. **f** Concentration of Cd in the residues after multiple FJH reactions. The dashed lines in (**e**, **f**) represent the starting contents and the approved World Health Organization (WHO) level for safe limits of agricultural soils.

Removal of toxic components is another major concern for e-waste processing^3,6,7,31^. The heavy metal removal capability of the FJH process was evaluated. Compared to precious metals, heavy metals, including Cr, Pb, Cd, As, and Hg, have much higher vapor pressures and lower boiling points (Fig. 4a and Supplementary Fig. 7b). Especially for the most toxic Cd, As, and Hg, the separation factors between them and precious metals could achieve ~10^5^ based on the theoretical analysis (Supplementary Note 4). The levels of heavy metals in PCB waste are in the range of 0.1–20 ppm (Fig. 4b). These values are above the safe limits of heavy metals in soils for agriculture as recommended by the world health organization (WHO)^32^. After one FJH, the heavy metal contents in the remaining solid (PCB-Flash) were greatly reduced (Fig. 4c). The removal efficiencies of Hg and Cd were calculated to be >80%, followed by Pb and As (>50%), and Cr (>35%) (Fig. 4d and Supplementary Note 2). These efficiencies were consistent with their vapor pressure values (Fig. 4a). The heavy metals were collected by condensation in the cold trap, as we did for the evaporative separation, and the collection yields were calculated (Fig. 4d). The collection yield matched well with the removal efficiency, demonstrating that most of the evaporated heavy metal was trapped by the cold trap, minimizing the leakage of heavy metals into the environment during the recycling process.

The concentration of heavy metals in the residue solids could be further reduced by multiple FJH reactions. After one FJH reaction, the concentration of Hg was reduced to below the safe limit of Hg in soils for agriculture (0.05 ppm) (Fig. 4e)^32^, the highest standard for waste disposal. As for Cd, three consecutive FJH cycles reduced the concentration to below the safe limit (0.003 ppm) (Fig. 4f)^32^. The concentration of As, Pb, and Cr were all reduced with an increase in the number of FJH reactions (Supplementary Fig. 20). Since each FJH only takes 1 s, multiple flashes are easily accomplished.

## Discussion

The proposed evaporative separation scheme is mainly targeted to the recovery of metals from e-waste. Nevertheless, it could exhibit the capability for the separation of metals. Theoretical calculation shows that large separation factors up to ~10^5^ could be realized for most metals with large vapor pressure differences (Supplementary Note 4, theoretical separation factors of the evaporative separation process based on the vapor pressure difference, Supplementary Fig. 21, Supplementary Table 3). The theoretical separation factors are calculated based on the vapor pressure of pure metals. They represent practical values for trace metal separation even with the melt alloy formation (Supplementary Note 4, the effect of melt alloy formation on the separation factors, Supplementary Fig. 22). The different recovery yields of precious metals (Fig. 1g) already demonstrated the separation feasibility of the FJH process based on the vapor pressure difference (Supplementary Note 4, the achieved separation ability by the evaporative separation, Supplementary Table 4). The chemical additives (Fig. 2a–f) also regulated the precious metals separation presumably due to their different chemical reactivity (Supplementary Note 4, the metal separation ability from the chemical additives, Supplementary Tables 5–7). The separation ability of the evaporative separation scheme could be further improved by progressively increasing the FJH temperature (Supplementary Note 4, the evidence-based predictions on the practices to increase the separation factors).

The cost and benefit of the FJH processing were evaluated since economic incentives are the main driver for waste recycling (Supplementary Note 5)^8^. FJH is a highly efficient heating process due to the ultrafast heating/cooling rate, the direct sample heating feature, and the short reaction duration, compared to traditional smelting furnaces where large amounts of energy are used to maintain the temperature of the whole chamber^33^. The FJH method has an energy consumption of ~939 kWh ton^−1^, which is ~1/500 of that for a lab-scale tubular furnace^34^, and ~1/80 of that for a commercially used Kaldo furnace in industrial scale^35^ (Supplementary Note 5). Hence, the FJH process for e-waste processing could have advantages over traditional pyrometallurgical processes.

The FJH process is scalable. According to the scaling rule revealed by the theoretical analysis, we could increase the FJH voltage and/or the capacitance of the capacitor bank when scaling up the sample mass (Supplementary Note 6 and Supplementary Figs. 23 and 24). By using a homemade automation system integrated with the FJH setup, our research lab has already realized a production rate of >10 kg day^−1^. Further commercial scaling up of the FJH process is underway (Supplementary Note 6). Considering the diminishing easily accessible ores of precious metals and the toxicity of several metal elements, the proposed FJH process to recover metals in e-waste could be a harbinger for near-future recovery methods.

## Methods

### Materials

CB (Cabot, Black Pearls 2000, average diameter 10 nm) was used as the conductive additive. The PCB waste was from a discarded computer. The PCB was cut into small pieces using a saw, and then ground into microscale powders by using a hammer grinder (Dade, DF-15). The salt additives were NaCl (J.T. Baker), NaF (Acros Organics), and NaI (Aldrich, 99.5%). The precious metals chlorides were RhCl_3_ (Aldrich, 38–40% Rh), PdCl_2_ (Aldrich, 99%), AgCl (Allied Chemical), and AuCl_3_ (Aldrich, >99.9%). Polytetrafluoroethylene (PTFE) powder was purchased from Runaway Bike. PVC, CPVC, and polyvinylidene fluoride (PVDF) plastic tubes from plumbing pipes were used as raw materials. The plastic waste products were cut into small pieces using a saw, and then ground into powders by using a hammer grinder (Dade, DF-15).

### FJH system and evaporative separation process

The electrical diagram of the FJH reactor is shown in Supplementary Fig. 3. There is a risk of electrocution so all safety measures should be obeyed carefully, as we listed in detail in the Supplementary Information. CB, PCB powders, and additives were mixed by using ball milling (MSE Supplies, PMV1-0.4 L). The reactants were loaded into a quartz tube with an inner diameter of 8 mm and an outer diameter of 12 mm. Copper wool was used as the porous electrode on one side to facilitate the gas diffusion, and a graphite rod was used as the electrode on the other side of the quartz tube. The tube was then loaded on the reaction stage and connected to the FJH system. The resistance was controlled by compressing the electrodes. The quartz tube was sealed by an O-ring. A vessel with a volume of ~40 mL was used as the cold trap. The vessel should withstand negative pressure (~10 Pa). A mechanical pump was used to pump the vessel to vacuum; then, the trap was immersed into the liquid N_2_ Dewar. This sequence must be followed to avoid O_2_ condensation in the N_2_ Dewar since O_2_ has a higher boiling point than N_2_. A capacitor bank with a total capacitance of 60 mF was charged by a direct current (DC) supply that can reach voltages up to 400 V. A relay with programmable ms-level delay time was used to control the discharge time. The high-voltage discharging brings the sample to a high temperature. The detailed conditions for the FJH are listed in Supplementary Table 1. For each condition, three FJH experiments were conducted to collect the total volatiles for sample digestion and ICP-MS measurement. Hence, the measured recovery yield is the average of three independent experiments using the same circuit board. After the FJH reaction, the FJH apparatus was allowed to cool to room temperature while the cold trap remained immersed in the liquid N_2_. Then, the trap was taken out from the liquid N_2_ while the apparatus remained under vacuum. After the trap warmed to room temperature, the vacuum was released.

### FJH under atmospheric and positive pressure

The FJH reaction is similar to the evaporative separation except with the following changes. The quartz tube was sealed by an O-ring to hold pressure. The porous Cu electrode side was connected to an inner gas (N_2_) cylinder by tubing that withstands pressure up to 5 bar. The pressure was adjusted to the desired values (1–4 atm) using a regulator and was monitored by a pressure gauge. Once the pressure was set, the FJH system was charged and then discharged for reaction. The detailed conditions for the FJH are shown in Supplementary Table 2. After the FJH reaction, the pressure was released, and the sample was removed for further analysis.

### Characterization

The SEM images were obtained by using a FEI Helios NanoLab 660 DualBeam SEM system at 5 kV. XRD was collected by using a Rigaku D/Max Ultima II system configured with a Cu Kα radiation (*λ* = 1.5406 Å). XPS spectra were taken using a PHI Quantera XPS system under the base pressure of 5 × 10^−9^ Torr. Elemental XPS spectra were collected using a step size of 0.1 eV with a pass energy of 26 eV. All of the XPS spectra were calibrated by using the standard C 1 *s* peak at 284.8 eV. STEM images and EDS maps were taken on a JEOL 2100 Field Emission Gun Transmission Electron Microscope under the voltage of 200 kV. TGA was conducted in air at a heating rate of 10 °C min^−1^ up to 1000 °C by using a Q-600 Simultaneous TGA/DSC from TA instruments. Calcination was conducted using the Mafu furnace in the air (NEY 6-160 A).

### Sample digestion and ICP-MS measurement

The standards were purchased from Millipore-Sigma. Three periodic table mixtures and Hg standard were used, where the composition is listed in Supplementary Table 8. HNO_3_ (67–70 wt%, TraceMetal^TM^ Grade, Fisher Chemical), HCl (37 wt%, 99.99% trace metals basis, Millipore-Sigma), and water (Millipore-Sigma, ACS reagent for ultratrace analysis) were used for sample digestion. The samples were digested by using a diluted aqua regia method^14,19^. The samples were soaked in HNO_3_/HCl (1 M each) solution at 45 °C for 24 h. The acidic solution was filtered to remove any undissolved particles. The solution was then diluted to the appropriate concentration range using 2 wt% HNO_3_ or HCl within the calibration curve. ICP-MS was conducted using a Perkin Elmer Nexion 300 ICP-MS system. The PCB raw powder, the condensed solid from the cold trap, the PCB-Flash powder, the PCB-Flash-Calcination powder, and the PCB-Calcination powder were leached using the same protocol.

## Supplementary information

Supplementary Information for Urban Mining by Flash Joule Heating

## Data Availability

The data supporting the findings of this study are available within the article and its Supplementary Information. Other relevant data are available from the corresponding author upon reasonable request. Source data generated in this study are provided in the Source Data file. The Source Data file is also uploaded to the Zenodo repository 10.5281/zenodo.5293916. Source data are provided with this paper.

## Source data

Source Data
