# Supplementary material for: Urban mining by flash Joule heating
Source: Nat Commun. 2021 Oct 4;12:5794. doi: 10.1038/s41467-021-26038-9 (PMC8490403; doi:10.1038/s41467-021-26038-9)
Supplement: Supplementary file 1 — Supplementary Information for Urban Mining by Flash Joule Heating [file 41467_2021_26038_MOESM1_ESM.pdf]

Supplementary Information for

Urban Mining by Flash Joule Heating

Bing Deng[1], Duy Xuan Luong[1], Zhe Wang[1], Carter Kittrell[1], Emily A. McHugh[1], and James M. Tour[1,2,3,4,*]

[1] Department of Chemistry, Rice University, Houston, Texas 77005, USA

[2] Smalley-Curl Institute, Rice University, Houston, Texas 77005, USA

[3] NanoCarbon Center and the Welch Institute for Advanced Materials, Rice University, Houston, Texas 77005, USA

[4] Department of Materials Science and NanoEngineering, Rice University, Houston, Texas 77005, USA

*Corresponding author. Email: tour@rice.edu

Supplementary Notes

Supplementary Note 1. The Numerical simulation.

The numerical simulation was conducted using the finite element software COMSOL Multiphysics 5.5.

Temperature simulation.

For the temperature distribution simulation, the Joule Heating mode in the AC/DC module was used with the following parameters (**Supplementary Fig. 6**):

(1) geometrical parameters: electrode radius (0.4 cm), electrode length (0.5 cm), materials radius (0.4 cm), materials length (2 cm).

(2) Materials parameters: electrical conductivity (0.2 S m^{-1}), and thermal conductivity (1 W m^{-1} K^{-1}).

(3) Boundary conditions: input voltage (150 V), ground (0 V).

Gas diffusion simulation.

In a typical FJH process, the vessel was first pumped to P_0 ~10 Pa. After a typical FJH, the pressure was P_1 = 12 kPa, hence the collected gas pressure was ΔP_1 = 12 kPa.

The volume of the vessel is V_1 ~40 mL, and the volume of the quartz tube is V_2 ~1 mL.

According to the Boyle's Law, **eq S1**:

$$V_1\Delta P_1 = V_2\Delta P_2 \quad \text{(S1)}$$

The inner pressure (ΔP_2) was calculated using **eq S2**:

$$\Delta P_2 = V_1\Delta P_1/V_2 = 480 \text{ kPa} \sim 5 \text{ atm.} \quad \text{(S2)}$$

Hence, the inner pressure generated during the FJH heating was set as 5 atm for the gas diffusion simulation.

For the gas diffusion simulation, the Laminar Flow mode was used with the following conditions (**Supplementary Fig. 18**):

(1) Geometrical parameters: reactor radius (0.4 cm), reactor length (2 cm), tube radius (0.1 cm), tube length (4 cm).

(2) Materials parameters: fluid (N_2) at temperature (273.25 K).

(3) Boundary conditions: inner pressure (500 kPa), outside pressure (0 Pa, 100 kPa, 400 kPa).

Supplementary Note 2. The calculation of recovery yield

For the evaporative separation, considering that the mass of PCB raw materials used for FJH is *m*(PCB), the concentration of precious metals in PCB raw materials was measured as *c*(PCB), and the mass of precious metals condensed in the cold trap was *M*(Gas), the recovery yield (Y(Gas)) was calculated by as in **eq S3**:

$$Y(Gas) = \frac{M(Gas)}{c(PCB)\times m(PCB)} \times 100\% \qquad \text{(S3)}$$

Alternatively, considering that the mass of remaining solid (PCB-Flash) after FJH was *m*(PCB-Flash), and the concentration of precious metals was *c*(PCB-Flash), the recovery yield could also be calculated in **eq S4**:

$$Y(Gas) = \frac{M(Gas)}{M(Gas)+c(PCB-Flash)\times m(PCB-Flash)} \times 100\% \qquad \text{(S4)}$$

In most cases, the two-calculation methods gave similar results because *c*(PCB) × *m*(PCB) = *M*(Gas) + *c*(PCB-Flash) × *m*(PCB-Flash). In this case, **eq S1** was used. However, in some cases, due to the inhomogeneous distribution of precious metals in PCB raw materials, the concentration of precious metals in the PCB raw materials used in different batches had some variation. This could result in a recovery yield >100% for the first method. In this case, **eq S2** was used to give a lower limitation of recovery yield.

For the FJH-improved leaching efficiency, considering that the mass of PCB raw materials used for FJH was *m*(PCB), the concentration of precious metals in PCB raw materials was measured as *c*(PCB), the mass of PCB-Flash solid was *m*(PCB-Flash), the concentration of precious metals in PCB-Flash solid was measured as *c*(PCB-Flash), then the recovery yield by leaching the PCB-Flash solid, Y(PCB-Flash), was calculated using **eq S5**:

$$Y(PCB - Flash) = \frac{c(PCB-Flash)\times m(PCB-Flash)}{c(PCB)\times m(PCB)} \times 100\% \qquad \text{(S5)}$$

Similarly, the recovery yield by leaching the PCB-Calcination, Y(PCB-Calcination), was calculated using **eq S6**:

$$Y(PCB-Calcination) = \frac{c(PCB-Calcination) \times m(PCB-Calcination)}{c(PCB) \times m(PCB)} \times 100\% \quad (S6)$$

where *m*(PCB-Calcination) was the mass of PCB-Calcination solid, the *c*(PCB-Flash) was the concentration of precious metals in PCB-Calcination solid.

The recovery yield by leaching the PCB-Flash-Calcination, Y(PCB-Flash-Calcination), was calculated using **eq S7**:

$$Y(PCB-Flash-Calcination) = \frac{c(PCB-Flash-Calcination) \times m(PCB-Flash-Calcination)}{c(PCB) \times m(PCB)} \times 100\% \quad (S7)$$

where *m*(PCB-Flash-Calcination) was the mass of PCB-Calcination solid, the *c*(PCB-Flash-Calcination) was the concentration of precious metals in PCB-Calcination solid.

For the removal of toxic heavy metals by FJH, considering that the mass of PCB raw materials used for FJH was *m*(PCB), the concentration of heavy metals in PCB raw materials was measured as *c*(PCB), the mass of PCB-Flash solid was *m*(PCB-Flash), the concentration of precious metals in PCB-Flash solid was measured as *c*(PCB-Flash), then the removal efficiency, Y(Removal), of toxic heavy metals by FJH was calculated using **eq S8**:

$$Y(Removal) = \frac{c(PCB) \times m(PCB) - c(PCB-Flash) \times m(PCB-Flash)}{c(PCB) \times m(PCB)} \times 100\% \quad (S8)$$

Furthermore, considering that the mass of toxic heavy metals collected by condensation in the cold trap was *M*(Gas), the collection yield, Y(Collection), was calculated using **eq S9**:

$$Y(Collection) = \frac{c(PCB-Flash) \times m(PCB-Flash)}{c(PCB) \times m(PCB)} \times 100\% \quad (S9)$$

Supplementary Note 3. The total composition analysis of the collected solid and evidence-based predictions on purification and refining.

The collected solids by the evaporative separation are a mixture of metals. There are already many commercial processes to separate individual metals from a mixture of metals. Here, we first did a complete composition investigation of metals (type and content) in the collected solids and provide evidence-based predictions on how to separate individual metals using readily accessible, well-established methods.

The total composition analysis of the collected solid.

We use the collected solid without chemical additive, and the collected solid with mixture halide additives (NaF, NaCl, and NaI) as representatives. The abundant metals in e-waste (Cu, Sn, Al, Fe, and Zn), the precious metals (Rh, Pd, Pt, Ag, and Au), and the toxic heavy metals (Hg, Cd, As, Pb, and Cr) were measured. Since the content of other metals are a few orders of magnitude less than those of the abundant metals, we think that the consideration of the above elements affords a reasonable approximation. The collected solid was totally digested and the ICP MS analysis of the metals was conducted. The total metal composition of the collected solids with and without the additives was shown in **Supplementary Fig. 14**. In both cases, the most abundant components are Cu with >60 wt%, followed by Al, Sn, Fe, and Zn with >1 wt%. For precious metals, the mass ratios were Ag, ~0.6 wt%; Pd, ~0.04 wt%; Rh, ~0.02 wt%; and Au, ~0.01 wt%. Cu is one major metal to be recovered with ~30% of total values for urban mining, so our FJH process also works for Cu recovery from e-waste.

The chemical states of precious metals in the collected solid.

Since the content of precious metals are <0.1 wt% (Supplementary Fig. 14), it is difficult to directly conduct the XPS analysis. Here, we added precious metal salts into the e-

waste and conducted the same FJH procedure to collect the volatiles for the XPS analysis. Specifically, $RhCl_3$, $PdCl_2$, AgCl, and $HAuCl_4$ were added into the mixture of e-waste and carbon black (CB) with a weight ratio of 5 wt% for each. After the same FJH process, the volatiles were collected and XPS measurements were conducted to analyze the chemical states of the precious metals. As shown in Supplementary Fig. 16, the Ag and Au were mainly in the elemental state. The minor peak at 85.2 eV for Au $4f_{7/2}$ could be from an Au-based metal alloy, for example, $AuIn_2$. For Rh and Pd, both elemental and oxidation states existed. The Rh $3d_{5/2}$ peak at 310.4 eV could be assigned to $RhCl_3$, and the Pd $3d_{5/2}$ peak at 338.0 eV could be assigned to $PdCl_2$. This difference might be attributed to the different chemical reactivity of the precious metals.

Evidence-based prediction on the purification and refining of precious metals.

There have been a few commercial processes for individual precious metals separation and refining, including selective precipitation, solvent extraction and solid-phase extraction[1,2]. The classical precipitation methods are based on the solubility difference of the ammonium salts of precious metal-chloro complexes[3]. The solvent extraction methods use solvent extractants to selectively extract a given metal and then transfer to the organic phase[4]. The solid-phase extraction relies on the use of ion-exchange resins with metal-selective ligands[5]. All these well-established techniques could be directly used in our collected solid for purification and refinement of individual precious metals.

Supplementary Note 4. Separation capability of the evaporative separation process.

Obtaining readily applicable pure metals from the complex e-waste usually relies on a lengthy engineering package, including beneficiation to purification. It usually has two major processes, the recovery of metal mixture from the e-waste raw materials, and the subsequent separation or refining of individual metals from the mixture. In commercial practices, after examination and beneficiation of the e-waste, the pyrometallurgical process (smelting) is applied to obtain a mixture solid of metals. Then the hydrometallurgical process (leaching) is used to obtain the leaching liquor with mixed metals. Finally, advanced refining process are used to separate and purify individual metals, with the main techniques including solvent extraction, leaching-precipitation, electro-oxidation and ion exchange[2].

In the manuscript, our proposed evaporative separation is first-of-all targeted on the separation of metals from the matrices (such as plastic, ceramics and carbon,) of e-waste. Such a high temperature (~3000 K), and assisted by the additives, could evaporate most of the metals. We did not seek to obtain the pure, individual metals. The evaporative separation scheme exhibits a capability for the separation of metals, which could be improved by further optimization of the FJH setup. We here first discuss the theoretical separation factor of metals based on the vapor pressure difference and the effect of alloy melt formation on the separation factors; second, we discuss the separation ability achieved now; third, we discuss the chemical additives assisted separation; fourth, we made evidence-based predictions regarding how to further improve the separation of the evaporative separation scheme.

Theoretical separation factors of the evaporative separation process based on the vapor pressure difference.

The e-waste contains most of the metals across the Periodic Table. Here, we choose the abundant metals in e-waste: Cu, Sn, Al, Fe, and Zn; the precious metals Rh, Pd, Pt, Ag, and

Au; and the toxic heavy metals Hg, Cd, As, Pb, and Cr, as representatives to calculate their separation factors. The vapor pressure-temperature relationships of these metals and C are plotted in **Supplementary Fig. 21a**. If we do not consider the alloy effect on the vapor pressure, the theoretical separation factors of these metals could be calculated based on the vapor pressure differences by **eq S10**,

$$\beta_{A-B} = \frac{P_A(t=t_{b,A})}{P_B(t=t_{b,A})} \quad \text{(S10)}$$

where $\beta_{A\text{-}B}$ is the separation factor of metal A and metal B, $P_A(t = t_{b,A})$ is the vapor pressure of metal A at the boiling point of metal A ($t_{b,A}$), and $P_B(t = t_{b,A})$ is the vapor pressure of metal B at $t_{b,A}$. The separation factors are listed in **Supplementary Table 3** and plotted in **Supplementary Fig. 21b**.

In most cases, the evaporative separation has a large separation factor of >100, demonstrating that the evaporative separation is a potential process for metal separation. The heatmap shows that the metals could be grouped into a few clusters, the group with low boiling points: Hg, As, Cd, Zn, and Pb; the group with median boiling points: Ag, Al, Cu, Sn, Cr, Au, Fe, and Pd; and the group with high boiling points: Rh and Pt. The heavy metals tend to have low boiling points and are the easiest to be removed from the e-waste, followed by the abundant metal groups, and then the precious metal groups. For the elements with large vapor pressure difference, the separation factor is large, *e.g.*, $\beta_{Ag\text{-}Pd} \sim 2439$; in contrast, the separation factor is small for the metals with similar evaporative behavior, *e.g.*, $\beta_{Cu\text{-}Al} \sim 1.25$.

The effect of melt alloy formation on the separation factors.

There might be alloy formation during the FJH process, yet it is not a certainty. In e-waste, the metals are usually separated by the matrix substrates, and the heating rate is ultrafast (>10^4 K s^{-1}) in the FJH process with short reaction duration (~1 s). As a result, the metals might not form alloys before their sublimation. Moreover, not all the metals will form a solid solution.

For example, Ag and Cu are thermodynamically immiscible, hence the melting will have no effect on their evaporative behaviors.

In the case of alloy melt formation, we discuss the partial vapor pressure of each metal components over alloys. For simplicity purposes, we consider the binary alloys AB. The equilibrium partial vapor pressure over liquid alloys is given by **eq S11**:

$$p_A = p_A^0 a_A \qquad \text{(S11)}$$

where p_{A} is the partial vapor pressure of metal A, p_A^0 is the vapor pressure of pure metal A, and a_{A} is the activity of component A in the alloy AB.

The activity has the following properties: (1) $a = 1$ for a pure phase that does not exhibit solid solution. Hence, for the metals that do not form alloys, like the case of Cu and Ag, the melting has no effect on their vapor pressure. (2) The activity is related to the mole fraction (x). In an ideal model, the activity equals to the mole fraction, $a_A = x_A$. In a non-ideal model, according to the normal activity-composition diagram (**Supplementary Fig. 22**), one component exhibits nearly ideal behavior at very low mole fractions or very high mole fractions. For such compositions, activity is approximately equal to mole fraction. In our cases, the precious metals and heavy metals are trace metals (~10 ppm level), and the major metals, such as Cu, Sn, and Al, are >1 wt% (>10000 ppm level) (**Supplementary Fig. 14**). Hence, the activity nearly equals the mole fraction for precious metals and heavy metals. In other words, the formation of solid melt will not have an apparent effect on their evaporative behavior. The calculated separation factors for Ag, Pd, Au, Rh, Pt, Cr, As, Pb, Hg, and Cd are generally not affected by the formation of alloy melt with abundant metals including Cu, Sn, Al, Fe, and Zn (**Supplementary Table 3**). (3) The activity is determined by the chemical potential of A in the alloy. For the components at comparable composition level, the activity would deviate from the mole fraction. In these cases, the separation factors should be corrected based on the activity. For example, Cu and Al are abundant metals in e-waste. There is literature reporting

the activity of the constituents in liquid Cu-Al alloy[6]. The separation factor of Cu and Al should be corrected accordingly. In our present work, we are targeting the separation of precious metals and heavy metals (as trace metals), hence we do not seek to calculate the precise separation factor for the abundant metals.

The achieved separation ability by the evaporative separation.

As shown in **Fig. 1g**, without the chemical additives, the recovery yields for the precious metals were Y(Rh) = 4.0%, Y(Pd) = 3.1%, Y(Ag) = 38.0%, and Y(Au) = 1.3%. These different recovery yield values demonstrate the separation ability of the FJH process (**Supplementary Table 4**). For example, the separation factor of Au and Ag is $\beta_{\text{Ag-Au}} \sim 29.2$. This value is comparable with the theoretical calculated separation factor of $\beta_{\text{Ag-Au, theory}} \sim 35$ (**Supplementary Table 3**).

The metal separation ability from the chemical additives.

In this manuscript, we demonstrate an improved recovery yield of precious metals by using the halide additives (**Figs. 2a-f**). The type of halide also affects the separation factors.

For example, by using NaCl as the additive, we achieved recovery yield of Y(Rh) = 25.2%, Y(Pd) = 17.5%, Y(Ag) = 75.8%, and Y(Au) = 0.3%. Accordingly, the separation factors are calculated (**Supplementary Table 5**). Similarly, by using NaF as the additive, we achieved recovery yield of Y(Rh) = 87.7%, Y(Pd) = 57.8%, Y(Ag) = 48.6%, and Y(Au) = 0.6%. By using NaI as additive, we achieved recovery yield of Y(Rh) = 39.6%, Y(Pd) = 41.7%, Y(Ag) = 42.7%, and Y(Au) = 63%. The separation factors by using NaF and NaI as the additives were calculated (**Supplementary Tables 6-7**). It is found that Cl works best for Ag, F works best for Rh and Pd, and I work best for Au.

Hence, the use of halides as additives could improve the recovery yield, while at the same time change the separation factors. The metal separation by the introduction of additives is attributed to the different chemical reactivity of precious metals with the chemical additives, which could be presumably further optimized for a better separation ability. For example, in future studies, we can first add Cl-containing additives to separate Ag, then F-containing additives to separate Rh and Pd, and then I-containing additives to separate Au. There is a tradeoff between recovery yield and separation ability; we focused on a high recovery yield in this manuscript and did not seek high separation ability.

The evidence-based predictions on the practices to increase the separation factors.

We noticed that the achieved separation factors are lower than the theoretically calculated ones (**Supplementary Tables 3-7**). To further increase the separation ability, we think that more carefully controlled temperatures and reaction duration would be helpful, which is the next step for the evaporative separation scheme. We are currently upgrading our FJH system with better temperature controllability. In the future, we presume to evaporate the metals one-by-one by progressively increasing the FJH temperature. We again note that there is always tradeoff between recovery yield and separation ability. Our current work mainly focused on the high recovery yield and hence put less effort toward the separation. Further work is essential to balance the recovery yield and metal separation.

Supplementary Note 5. The energy consumption and cost evaluation

The energy consumption was calculated using **eq S12**:

$$E = \frac{(V_1^2 - V_2^2) \times C}{2 \times M} \qquad \text{(S12)}$$

Where E is the energy per gram (kJ g^{-1}), V_1 and V_2 are the voltage before and after flash Joule heating, respectively, C is the capacitance (C = 60 mF), and M is the mass per batch.

For a typical trial, V_1 = 150 V, V_2 = 0 V, and M = 0.2 g, the energy was calculated to be:

E = 3.38 kJ g^{-1} = 9.39 × 10^{-4} kWh g^{-1} = 939 kWh ton^{-1}

Given that the industrial price of electric energy in Texas, USA is \$0.02 kWh^{-1}, the cost for treatment of 1 ton of e-waste would be P = \$18.78 ton^{-1}.

As a reference at the laboratory scale, Balaji *et al.* conducted a cost-benefit analysis on metal recovery from PCB using pyrometallurgy using a tubular furnace[7]. For the PCB mass of 200 g, they reported the electrical consumption of 100 kWh. This corresponds to 5 × 10^5 kWh ton^{-1}, or \$$10^4$ ton^{-1}. The cost of the FJH process is 939 kWh ton^{-1} or \$18.78 ton^{-1}, corresponding to ~1/500 of that of the tubular furnace. In an industrial-scale case, Boliden Ltd. Ronnskar Smelter, Sweden used a Kaldo furnace for smelting[8]. The e-waste was converted into a mixed Cu alloy by the Kaldo furnace, which is similar to our collected solids by evaporative separation using the FJH setup. They reported the required energy of 274 GJ ton^{-1} for e-waste processing. While in our case, the energy consumption is 3.38 GJ ton^{-1}, corresponding to ~1/80 of the Kaldo furnace. We note that the energy consumption is optimized for the commercial Kaldo furnace, and we presume the energy consumption of our FJH setup could be further reduced when scaling up to industrial scale.

Supplementary Note 6. Strategy for scaling up of the FJH process.

Joule heating is a mature heating technique and has been widely used in multiple practical devices and industrial processes, for example, electric fuses and electric heaters. The FJH disclosed here is intrinsically a Joule heating process. The difference of the FJH and conventional Joule heating technique lies in the modes of electrical energy supply and the heating duration. The conventional Joule heating process uses direct current (DC) or alternating current (AC) sources to provide a stable electric output. For our FJH process, a capacitor bank is used to provide a pulsed voltage output in a short time (down to ms scale). The FJH process is indeed highly scalable. Here, we first conduct the theoretical analysis on the scaling rule of the FJH process; second, we mention the batch-by-batch scaling up experiments with productivity up to kg scale in our research lab; third, we make an evidence-based prediction on how the FJH process could be scaled up by a continuous, roll-to-roll manner; fourth, we briefly discuss the undergoing industrial-scale application of the FJH process.

The scaling rule of FJH process revealed by theoretical analysis.

The *accessible high temperature* and the *uniform temperature distribution* across the sample are the two key points when scaling up the FJH process. For Joule heating, the heat amount (Q) is determined by **eq S13**,

$$Q = I^2 R t \quad \text{(S13)}$$

where I is the current passing through the sample, R is the resistance of the sample, and t is the discharging time. We then consider the heat per volume (Q_v) determined by **eq S14**,

$$Q_v = j^2 \rho_e t \quad \text{(S14)}$$

where j is the current density, ρ_e is the electrical resistivity, and t is the discharging time.

The temperature is proportional to the heat since the heat capacity of the sample is constant. Since the electrical resistivity of the sample is constant, to maintain a constant Q_v and t when scaling up the sample, we need to maintain a constant j.

The charge (q) in the capacitor bank is determined by **eq S15**,

$$q = CV \quad \text{(S15)}$$

where C is the total capacitance, and V is the charging voltage. If we suppose the charges in the capacitor bank are discharged in the discharging time of t, the current (I) passing through the sample is calculated by **eq S16**,

$$I = \frac{q}{t} \quad \text{(S16)}$$

Then, the current density (j) is determined by **eq S17**,

$$j = \frac{I}{S} = \frac{CV}{St} \quad \text{(S17)}$$

where S is the cross-sectional area of the sample. Considering the cylinder-shaped sample (which is usually the case), the sample mass (m) is calculated by **eq S18**,

$$m = \rho_m SL \quad \text{(S18)}$$

where ρ_m is the density of the sample, S is the cross-sectional area of the sample, and L is the length of the sample.

Above all, we obtain a formula determining the current density of **eq S19**,

$$j = \frac{CV\rho_m L}{mt} \quad \text{(S19)}$$

As discussed, to scale up the sample mass (m), we need to maintain a constant current density. This could be realized by the measures of (1) increasing the FJH voltage (V), and/or (2) increasing the capacitance (C).

In our FJH setup, we use a commercial aluminum electrolytic capacitor (450 V, 6 mF, Mouser #80-PEH200YX460BQU2). The state-of-art commercial aluminum electrolytic capacitor has the maximum rated voltage of V_1 = 630 V, and capacitance of C_1 = 2.7 F. In our

typical experiment, we use a FJH voltage of V_0 = 150 V and C_0 = 0.06 F for the FJH of sample with mass of m_0 = 0.2 g. According to eq S18, by using just one state-of-art capacitor, the mass is $m_1 = m_0\ (C_1\ V_1)/(C_0V_0)$ = 37.8 g per batch. The capacitors could be connected in parallel to get a high total capacitance by **eq S20**,

$$C_{total} = C_1 + C_2 + \cdots + C_n \qquad \text{(S20)}$$

Considering that we use a capacitance bank composed of 30 aluminum capacitors with the total capacitance of C_{total} = 81 F, the mass will be m_{batch} = 1.1 kg per batch in the discharging time of 1 s. The re-charging of the capacitor bank is the slowest step of the process, which could be compensated by a high-speed charging technique. Supposing the total time is t_{total} = 10 s per batch, one such FJH setup could process the e-waste of m ~ 10 tons per day. Based on our experience and these calculations, it is reasonable to conclude that the FJH process is highly scalable, with the capability for industry-scale application.

The demonstration of the scaling of the FJH process in our research lab.

In our typical experiment, we use a mass of m_0 = 0.2 g, with the FJH condition of V_0 = 150 V and C_0 = 0.06 F. Here, we demonstrate the scaling up of FJH to a scale with mass of m_1 = 2 g and m_2 = 4 g per batch (**Supplementary Fig. 23a**). We used a capacitor bank composed of 104 aluminum capacitors (6 mF, 450 V, Mouser #80-PEH200YX460BQU2) in parallel, so the total capacitance is C_1 = 0.624 F. For the sample mass of m_1 = 2 g, we use a FJH voltage of V_1 = 150 V, and for the sample mass of m_1 = 2 g, we use a FJH voltage of V_2 = 300 V, thus these conditions fit with the scaling rule of **eq S19**. Since temperature is a key parameter for the e-waste processing in our evaporative separation scheme, we recorded the temperature for those samples (**Supplementary Figs. 23b-d**). It is found that the temperature reaches >3000 K for all the samples, demonstrating the effective scaling up of the FJH process.

The evidence-based prediction of the continuous processing of e-waste by the FJH process.

In addition to the batch-by-batch process, we made evidence-based predictions for the continuous processing mode of the FJH processing of e-waste. As shown in **Supplementary Fig. 24**, two baffles separate the feeds and the remaining solid. After the FJH, the bottom baffle is opened and the remaining solid is removed from the reactor. The top baffle is then opened, and the feeds are loaded into the reactor for the next FJH reaction. Since the collected volatiles in the cold trap are very little per FJH, it is not necessary to change the collection vessel. Note that this is only one possible continuous production method. Many mature engineering practices could be applied.

Industrial-scale application of the FJH process is underway.

The FJH process, which was invented by our group for the synthesis of flash graphene[9], is already undergoing industrial-scale scaling up by Universal Matter, Inc (https://www.universalmatter.com/). The equipment and process developed and optimized for the flash graphene synthesis are ready to be shifted for the processing of e-waste. The evaporative separation system consisted of the FJH and the gas collection setups. The gas collection setup uses a cold trap with a mild vacuum, which should be scalable by using a larger vessel and mechanical pump.

Supplementary Figures

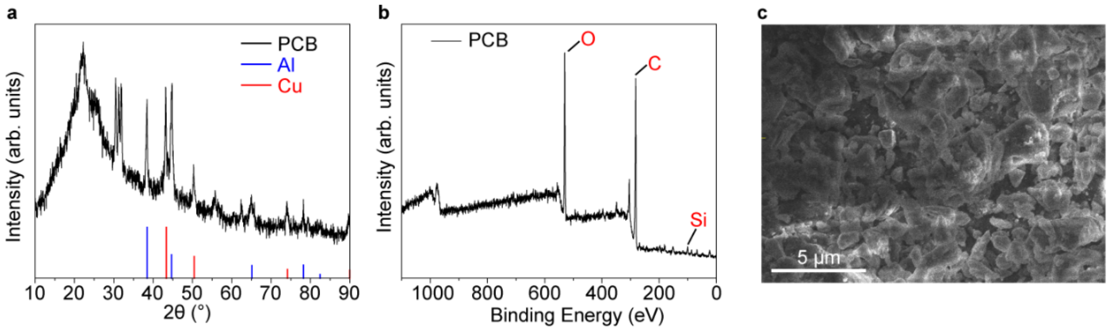

Supplementary Fig. 1 | Characterization of the printed circuit board (PCB) powder. (a) X-ray diffraction (XRD) of the PCB powder. Al and Cu are abundant metals in PCB raw materials. (b) X-ray photoemission spectroscopy (XPS) full spectrum of PCB powder. (c) Scanning electron microscopy (SEM) of the PCB powder after mechanical hammer grinding.

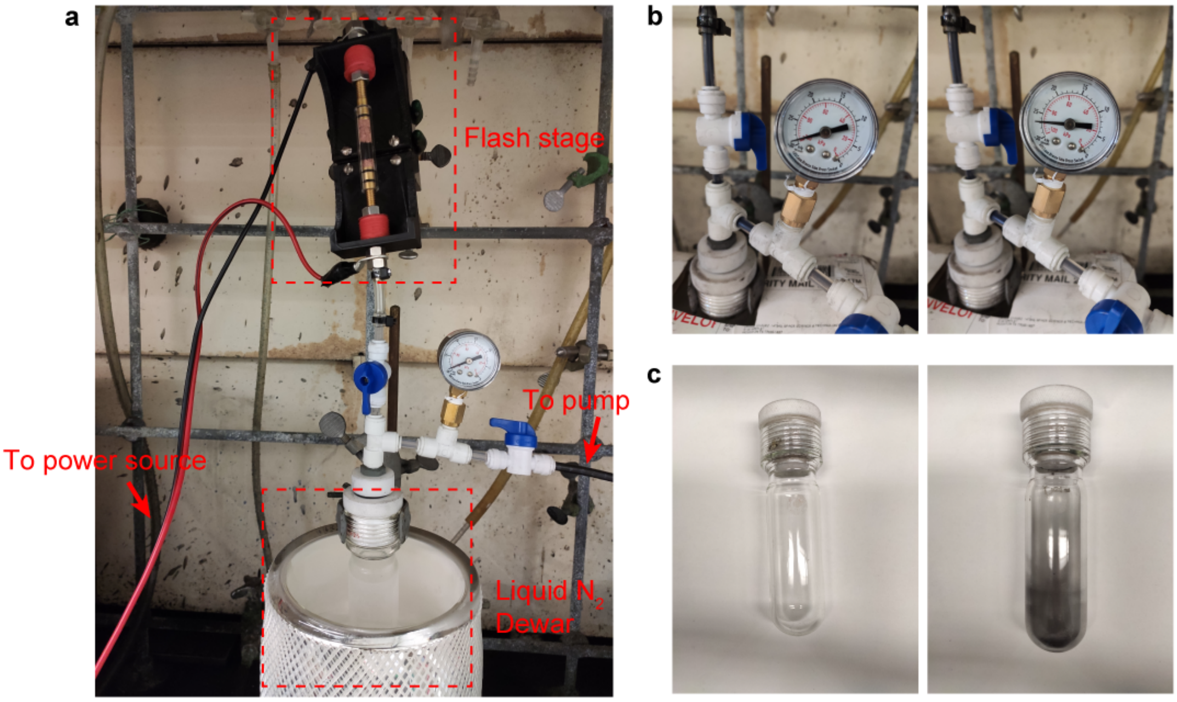

Supplementary Fig. 2 | Picture of the system to collect the evaporated metal vapor. (a) Picture of the evaporative collection system. (b) Picture of the vacuum gauge before and after flash Joule heating (FJH). (c) Picture of the condensate vessel before and after the FJH reaction.

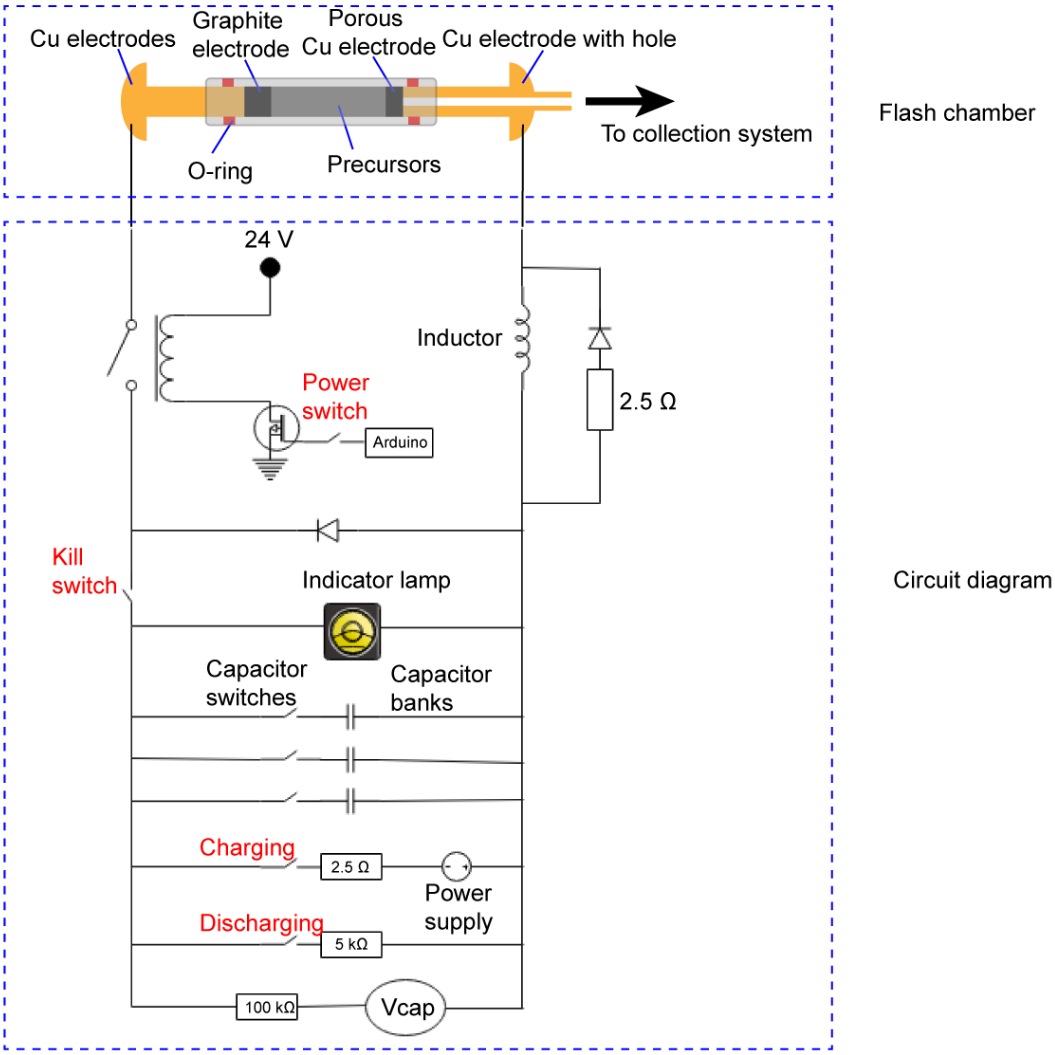

Supplementary Fig. 3 | Electrical circuit diagram of the flash Joule heating (FJH) system. The total capacitance of the capacitor bank is 60 mF. More details about the electrical components could be found in our previous publications[9].

CAUTION: There is a risk of electrical shock if improperly operated. Safety glasses for welding are recommended to block the infrared and ultraviolet light during the flashing reaction. There are numerous safety measures built into the system. But, as a final level of caution, the "one hand rule" should be obeyed (do not touch the apparatus with both hands, preventing the closing of a circuit through your body if anything goes wrong), and thick rubber

gloves that extend to the elbows should be used. More safety implementations can be found in our previous publications[9-11], which we also listed below.

Safety guidance of the FJH process.

1. Enclose or carefully insulate all wire connections.
2. All connections, wires, and components must be suitable for the high voltages and currents.
3. One hand rule. Use only one hand when working on the system, with the other hand not touching any grounded surface.
4. Provide a mechanical discharge circuit breaker switch connected to a power resistor of a few hundred ohms to rapidly bleed off the capacitor charge.
5. Provide a “kill” circuit breaker switches to disconnect the sample holder from the capacitor bank.
6. Post high voltage warning signs on the apparatus.
7. Keep in mind that the system can discharge many thousands of Joules in milliseconds, which can cause components such as relays to explore.
8. Keep a voltmeter with high voltage test leads handy at all times. When working on the capacitor bank, always check the voltage on each.
9. Wear thick rubber gloves when using the apparatus to protect from electrocution.
10. All users should be properly trained by an experienced electrical technician.

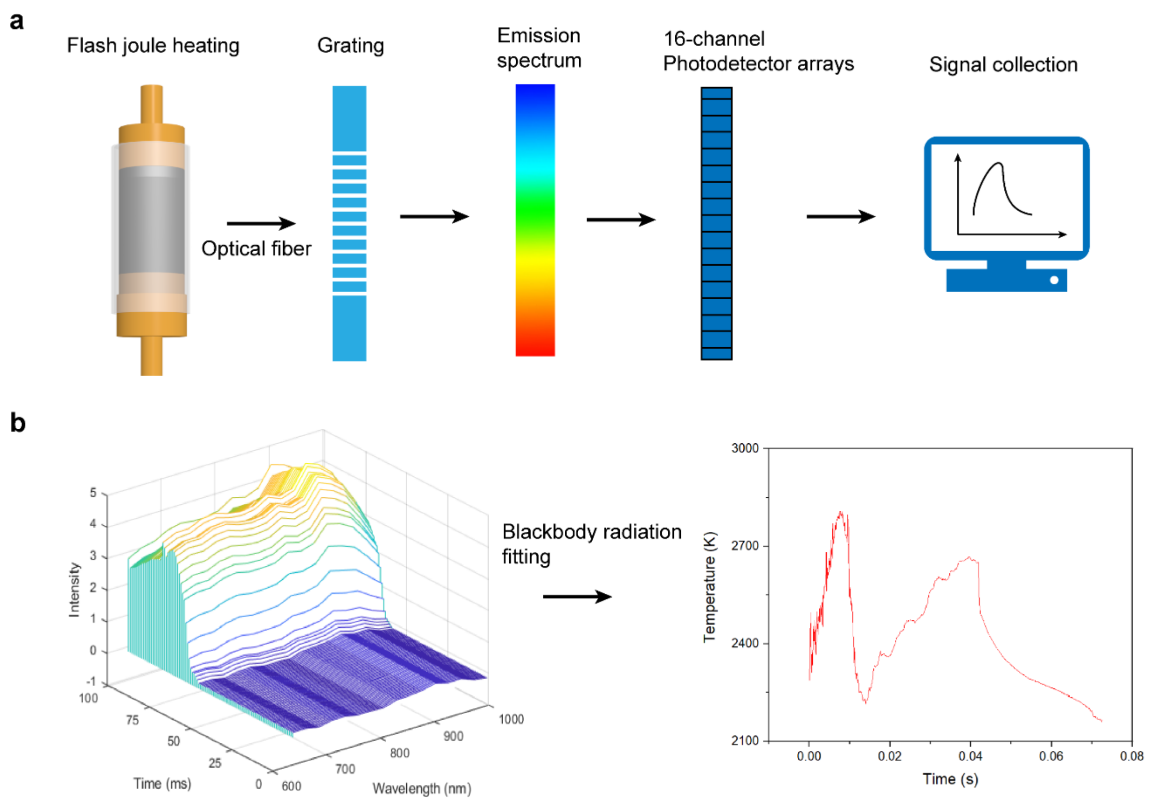

Supplementary Fig. 4 | Time-resolved temperature measurement by fitting the blackbody radiation. (a) Schematic configuration of the time-resolved pyrometer for spectrum collection during the flash Joule heating (FJH) process. Black body radiation from the sample during FJH is collected by an optical fiber and separated by a customized grating black box. The spectrum radiance was recorded using a 16 pixels photodiode array (Hamamatsu S4111-16R) at 600 – 1100 nm. The reversed bias voltages from the photodiode arrays are collected by a National Instrument multifunction I/O device PCIe-6320. (b) Temperature determination by fitting of the black body radiation. The spectra were fitted to the blackbody radiation equation to acquire a time-resolved temperature of the sample. Prior to measurement, the temperature was calibrated by using a 2800 K lamp.

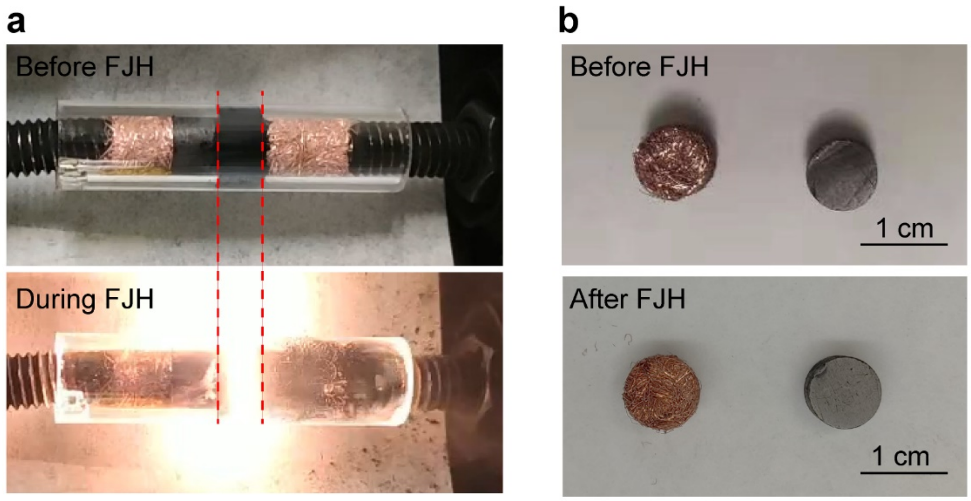

Supplementary Fig. 5. Durability of the flash Joule heating (FJH) setup. (a) Image of the sample before and during FJH. (b) Images of the porous Cu electrode (on the left in both photos) and graphite electrode (on the right in both photos) before and after FJH.

The FJH process could achieve a high temperature, but the high-temperature region is limited to the sample. According to the Joule heating formula, $Q = I^2Rt$, the heat amount is proportional to the resistance. The resistance values of the Cu electrode, the graphite electrode, and the sample are ~0.09 Ω, ~0.11 Ω, and >2.0 Ω, respectively. The Cu and graphite electrodes have much higher conductivity than the sample. Hence, the voltage drop was mainly imposed on the sample, and the heat amount generated by the discharging mostly retains on the sample. During the FJH, the strong light emission region is limited to the sample (**Supplementary Fig. 5a**), indicating that Cu and graphite electrodes remain low temperature. The high thermal conductivity of the Cu electrodes also helps the fast thermal dissipation and prevents the high temperature. Moreover, the FJH time is very short, with the >3000 K temperature in tens of ms. The Cu electrode and graphite rod show no obvious change after the FJH other than the contamination of the Cu electrode by CB (**Supplementary Fig. 5b**). The resistance of the Cu

electrodes and the graphite electrodes remains the same after the FJH. Hence, the FJH process has no significant effect on the Cu and graphite electrodes.

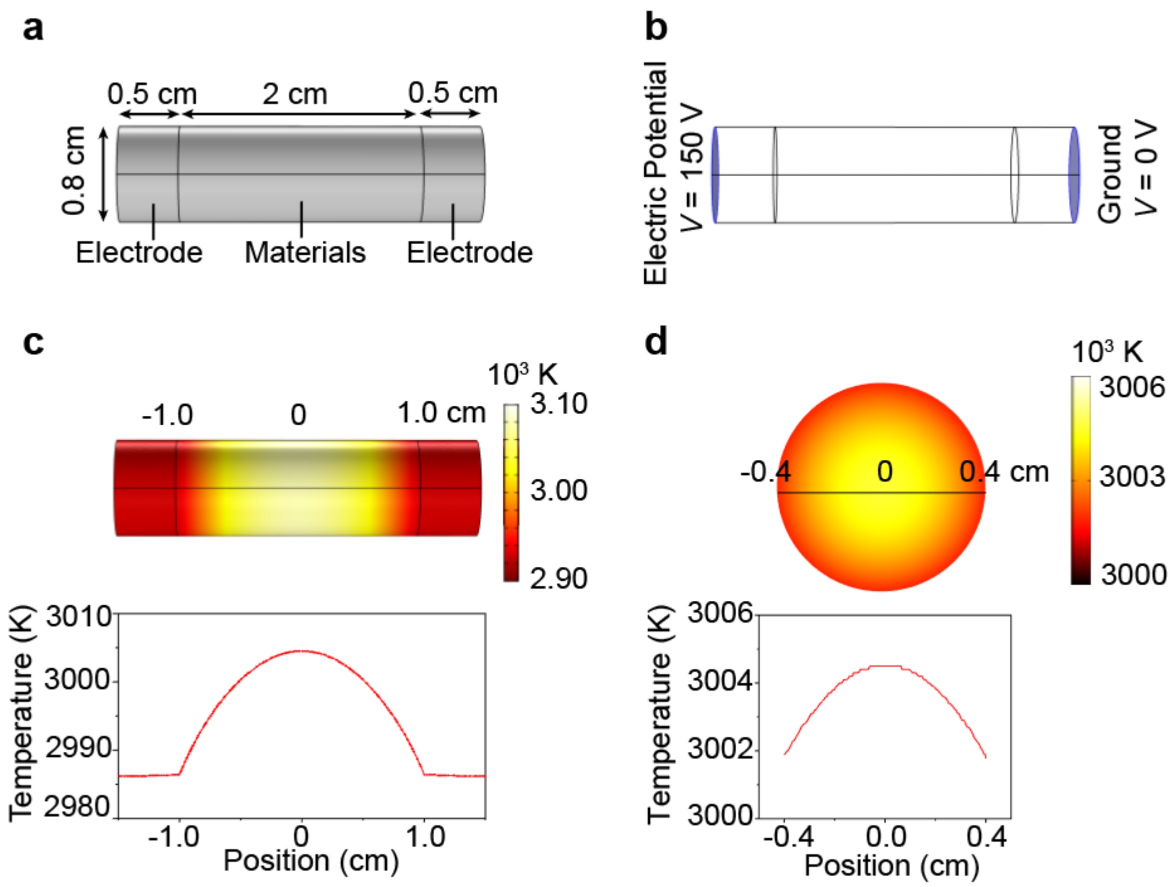

Supplementary Fig. 6 | Temperature simulation. (a) Geometrical parameters. (b) Boundary conditions. (c) Temperature distribution and the temperature profile of the sample along the longitudinal direction. (d) Temperature distribution and the temperature profile of the sample along the radial direction. The results show that the flash Joule heating (FJH) is homogenous both in the longitudinal and the radial directions.

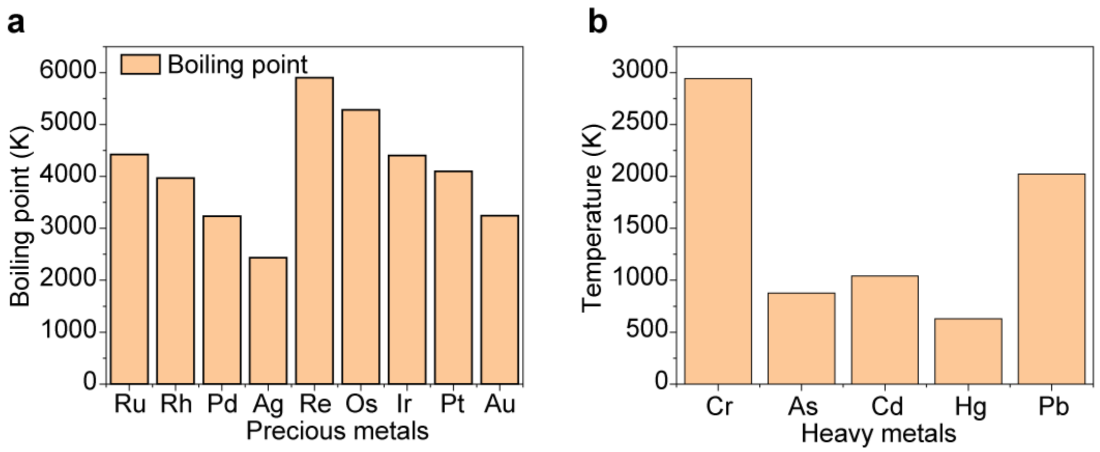

Supplementary Fig. 7 | The boiling points of (a) precious metals and (b) heavy metals.

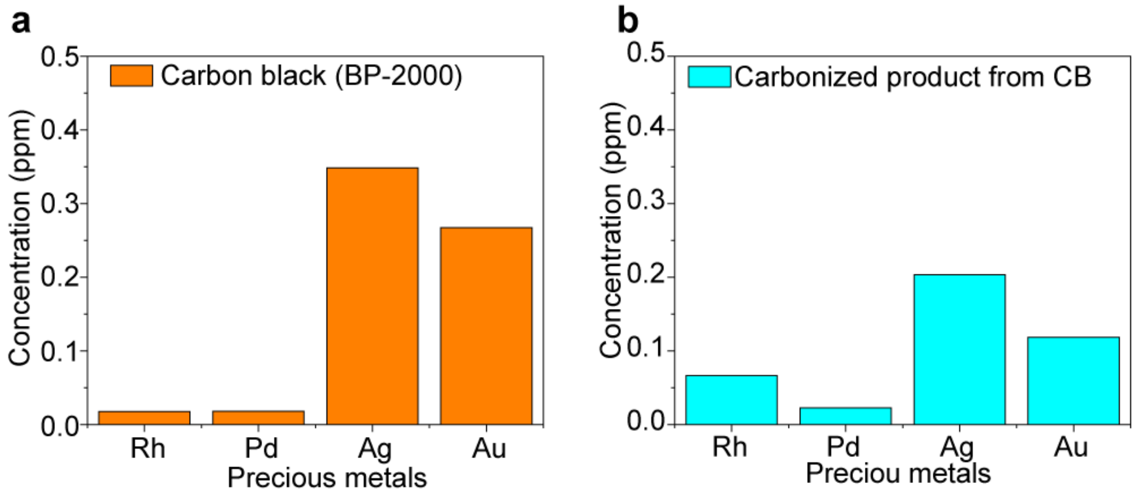

Supplementary Fig. 8 | Precious metals concentration in carbon black (CB). (a) Precious metals concentration in CB (BP-2000). (b) Precious metals concentration in the carbonized product obtained from flash Joule heating (FJH) of CB (BP-2000). The concentration of precious metals in CB are ~1 – 2% of those in printed circuit board (PCB).

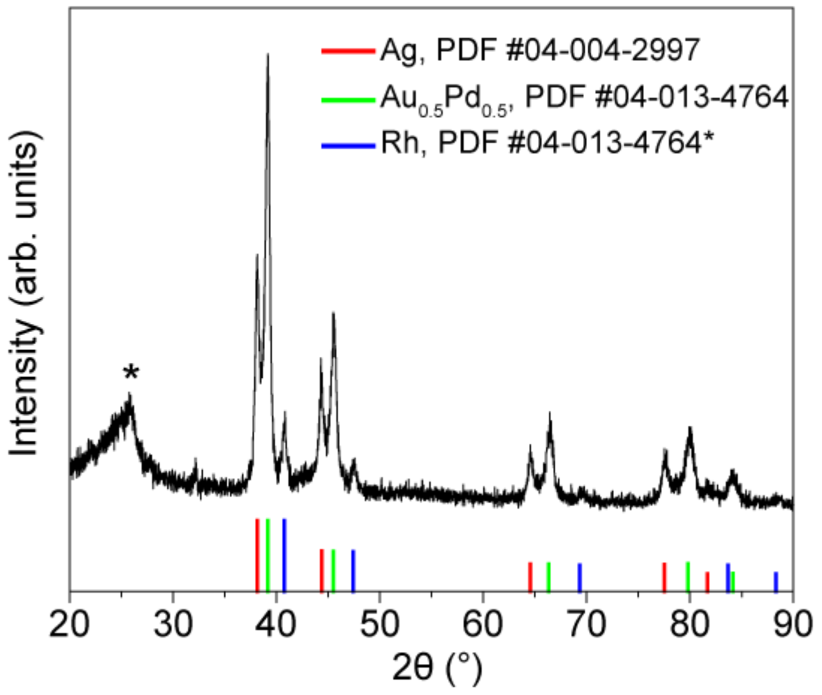

Supplementary Fig. 9. X-ray diffraction (XRD) pattern of the mixture of $RhCl_3$, $PdCl_2$, AgCl, $HAuCl_4$ and carbon black (CB) after flash Joule heating (FJH). The peak (*) denotes the graphite (0002) due to the graphitization of the CB.

The precious metals as the late transition group metals usually have weak affinity with C and almost no solubility for carbon. The precious metals tend to not form carbide phases even at high temperature[12]. For example, there is no experimental evidence for a possible inorganic crystalline gold carbon compound. Experimentally, we mixed $RhCl_3$, $PdCl_2$, AgCl, and $HAuCl_4$ with CB (5 wt% for each) and conducted the FJH. The XRD pattern of the product is shown in **Supplementary Fig. 9**. The XRD result showed that there were pure metal phases and metal alloy phases. No precious metal carbide phase was observed. Hence, the use of CB as conductive additives will not affect the evaporative behavior of precious metals.

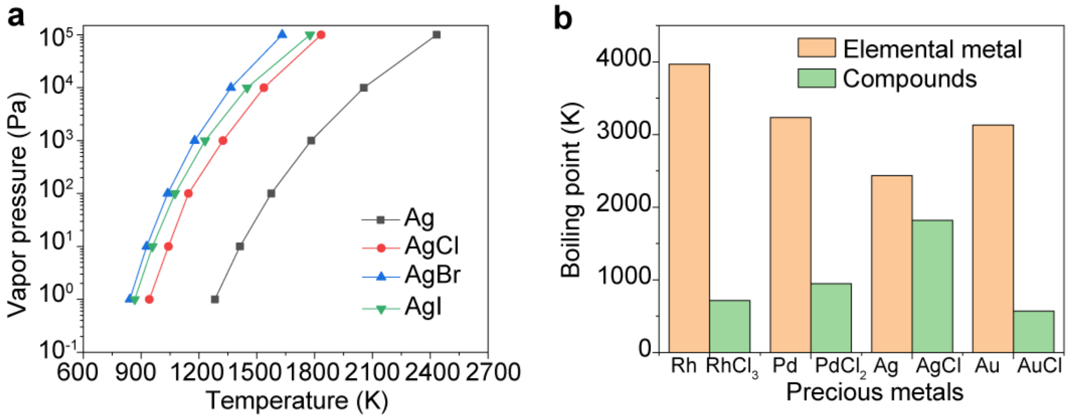

Supplementary Fig. 10 | Vapor pressure and boiling point of metal halides. (a) Vapor pressure-temperature relationships of Ag and Ag halides[13]. (b) Boiling points of elemental metals and metal chlorides[13]. Note that the $PdCl_2$ boiling point is the decomposition temperature.

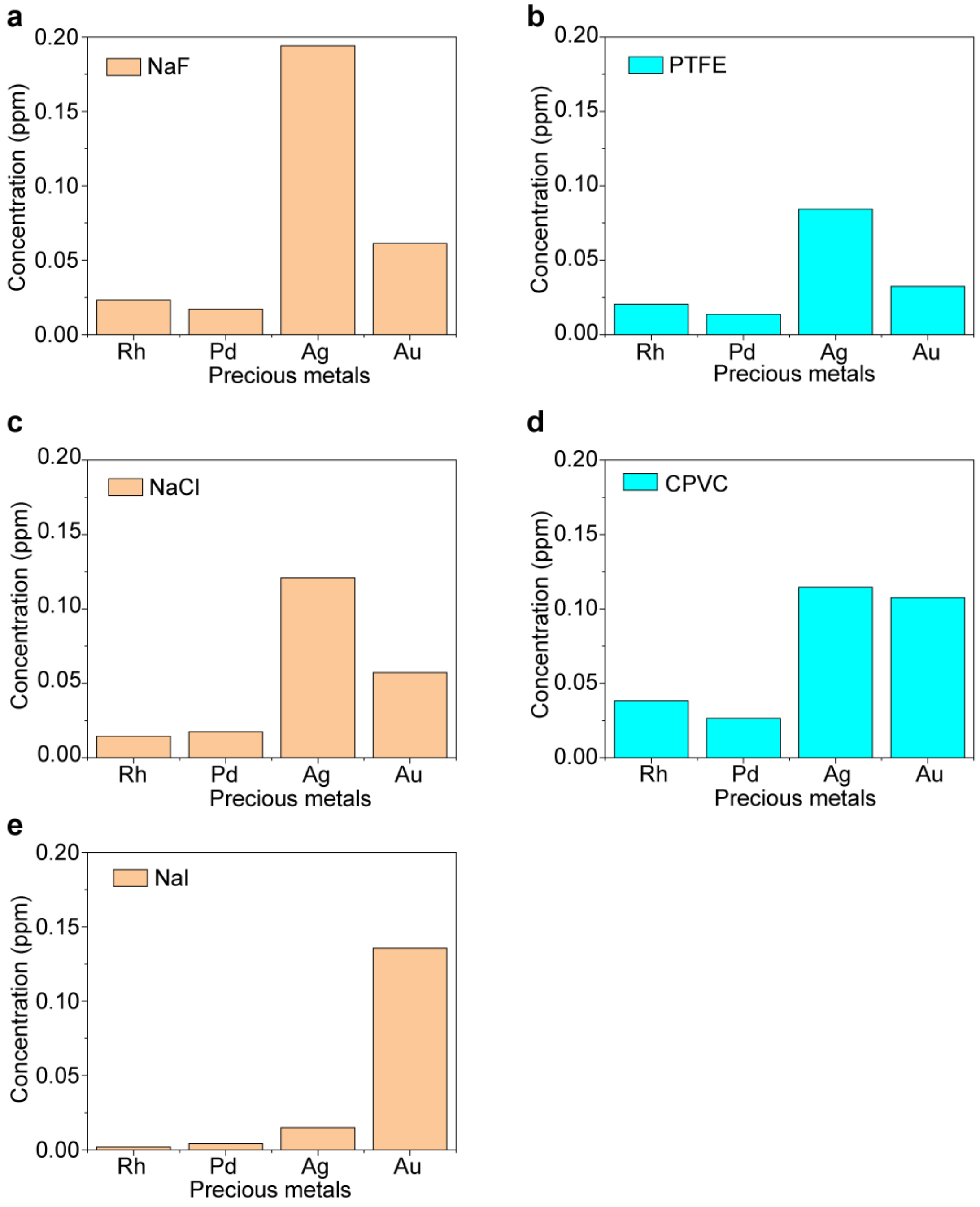

Supplementary Fig. 11 | Concentration of precious metals in additives. (a) NaF. (b) PTFE. (c) NaCl. (d) CPVC. (e) NaI. The concentration of precious metals in those additives are <1% of those in printed circuit board (PCB) raw materials.

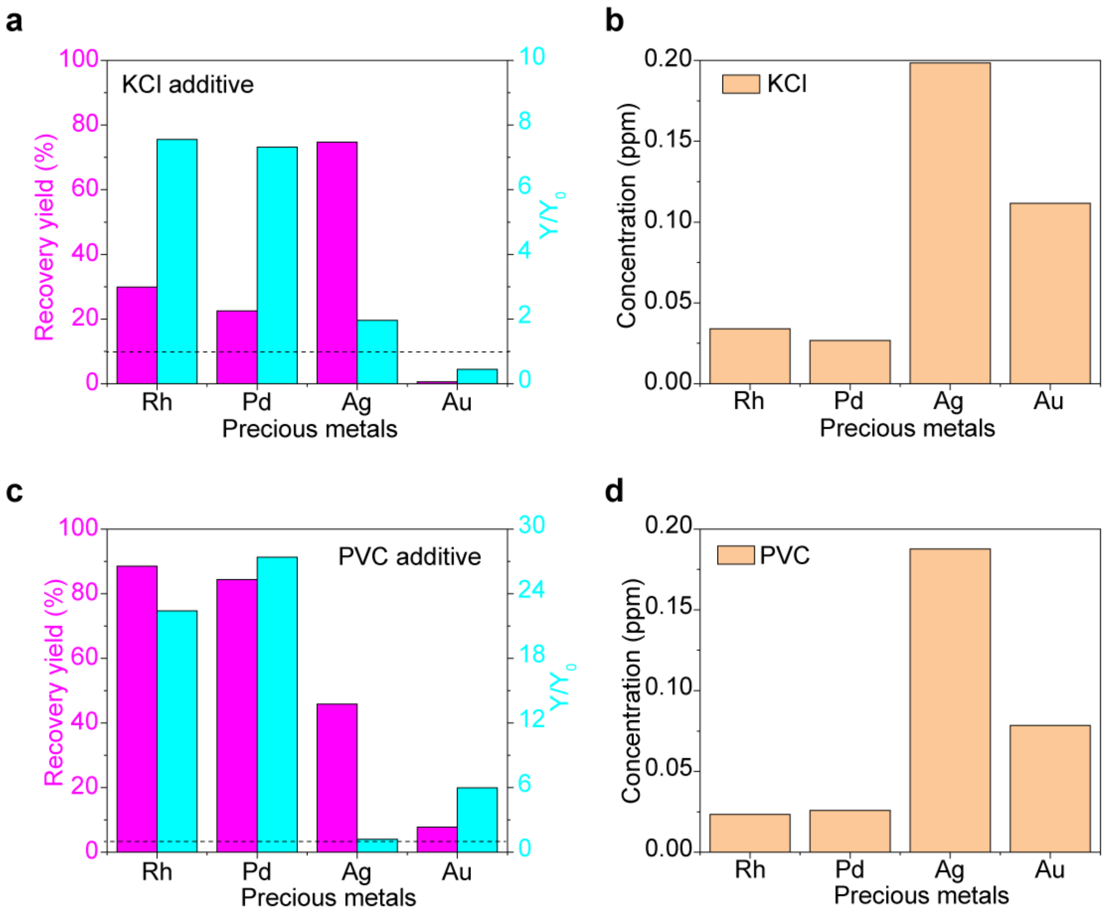

Supplementary Fig. 12 | Recovery yield improvement by using chloride additives. (a) Increase in recovery yield using KCl additive. (b) Concentration of precious metals in KCl. (c) Increase in recovery yield by using polyvinyl chloride (PVC) additive. (d) Concentration of precious metals in PVC. Care should be taken with these metal salts since carbothermal reduction to the metal can occur to afford metal(0) that can combust in water or moist air, though we never observed that problem at this scale.

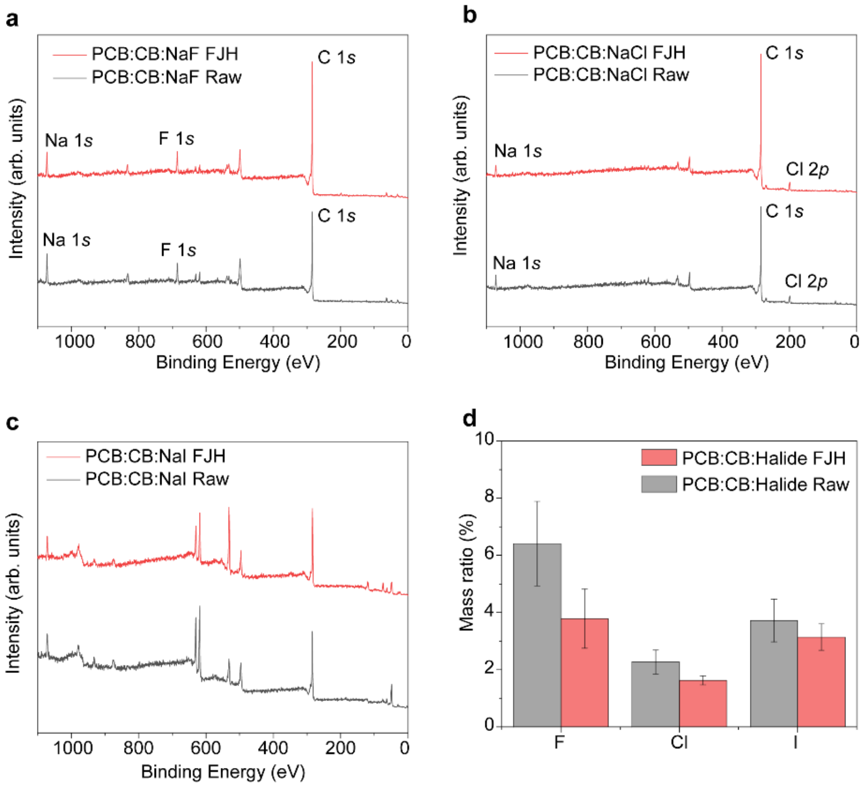

Supplementary Fig. 13. The evaporative loss of halides during the flash Joule heating (FJH) process. (a) X-ray photoemission spectroscopy (XPS) full spectra of the sample with NaF additives before and after FJH. (b) XPS full spectra of the sample with NaCl additives before and after FJH. (c) XPS full spectra of the sample with NaI additives before and after FJH. (d) The mass ratios of halide additives before and after FJH.

The mass ratios of halide in the raw materials were F, ~6.4%; Cl, ~2.3%; and I, ~3.7%. After FJH, the halide content in the remaining solids was F, ~3.8%; Cl, ~1.6%; and I, ~3.1%. This corresponds to the evaporative loss of F, ~40%; Cl, ~30%; and I, ~16%. The halide salts are expected to be easily recovered by a water washing and precipitation process due to their high solubility, while the components of the e-waste and carbon have low water solubility if any. Hence, it is possible to recovery the halides either remaining in the solids or evaporated and collected in the cold trap. The use of the halides will not introduce significant additional materials cost.

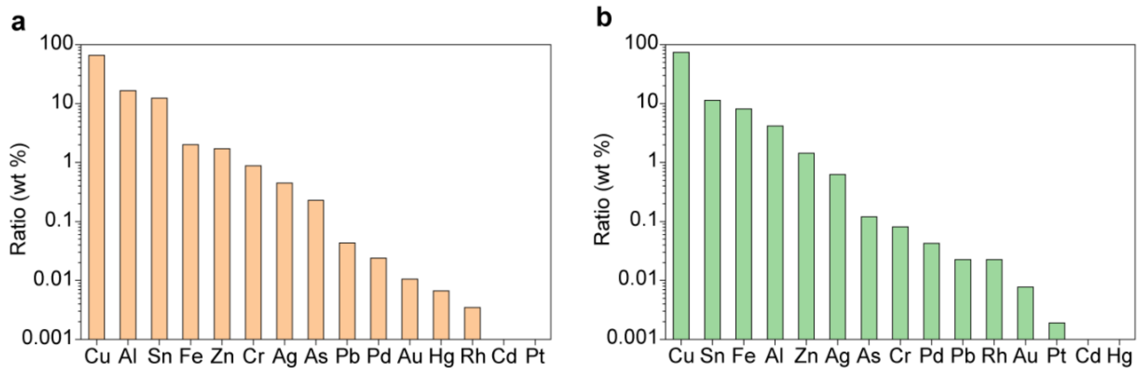

Supplementary Fig. 14. Total composition analysis of the collected solid. (a) Metal mass ratios of 15 elements in the collected solid without additive. (b) Metal mass ratios of 15 elements in the collected solid with the mixture additives NaF, NaCl, and NaI.

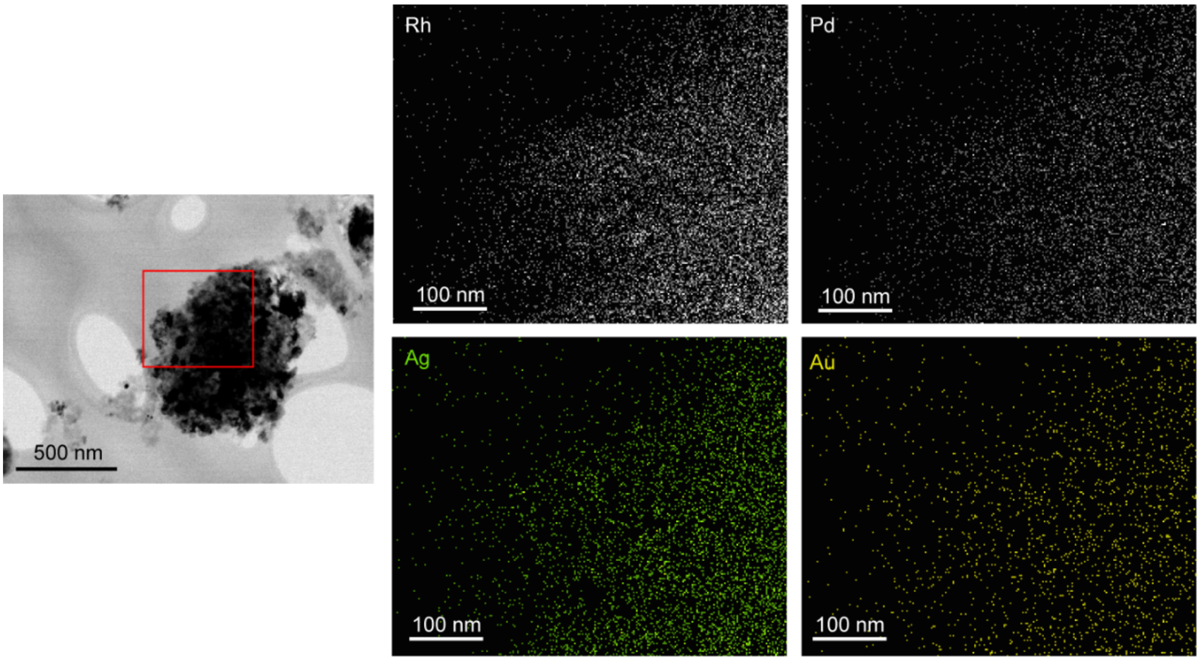

Supplementary Fig. 15 | Scanning transmission electron microscopy (STEM) image of the collected solid and Energy dispersion spectroscopy (EDS) maps of Rh, Pd, Ag, and Au. The element maps prove that the successful collection of precious metals. The metals spread over the entire collected solid.

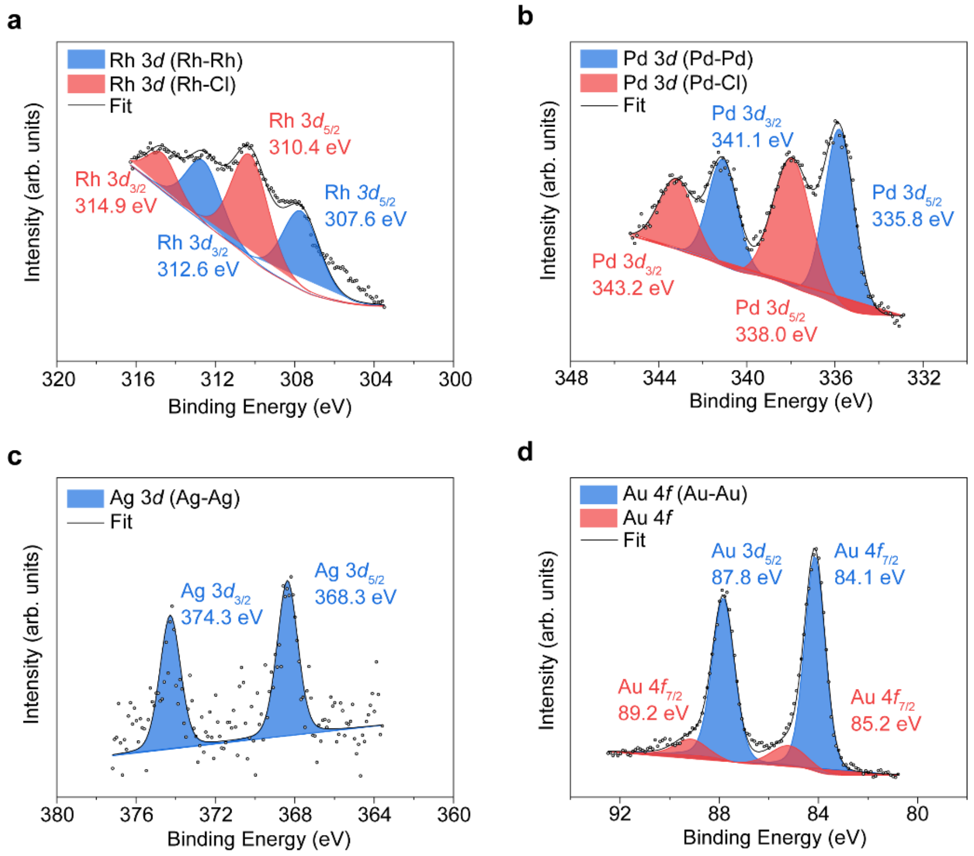

Supplementary Fig. 16. Chemical state analysis of the collected solids. (a) X-ray photoemission spectroscopy (XPS) fine spectrum of Rh. (b) XPS fine spectrum of Pd. (c) XPS fine spectrum of Ag. (c) XPS fine spectrum of Au.

Since the contents of precious metals are <0.1 wt% (**Supplementary Fig. 14**), it is difficult to directly analyze by XPS. Here, we added $RhCl_3$, $PdCl_2$, AgCl, and $HAuCl_4$ into the mixture of e-waste and CB (5 wt% for each) and conducted the same FJH process and collected the volatiles for XPS analyses. The XPS fine spectra for precious metals were shown in **Supplementary Fig. 16**. The Ag and Au were mainly in the elemental state. The minor peak at 85.2 eV for Au $4f_{7/2}$ could be from the Au-based metal alloy, for example, $AuIn_2$[14]. For Rh and Pd, both elemental and oxidation states existed. The Rh $3d_{5/2}$ peak at 310.4 eV could be assigned to $RhCl_3$[15], and the Pd $3d_{5/2}$ peak at 338.0 eV could be assigned to $PdCl_2$[16]. This difference might be attributed to the different chemical reactivity of the precious metals.

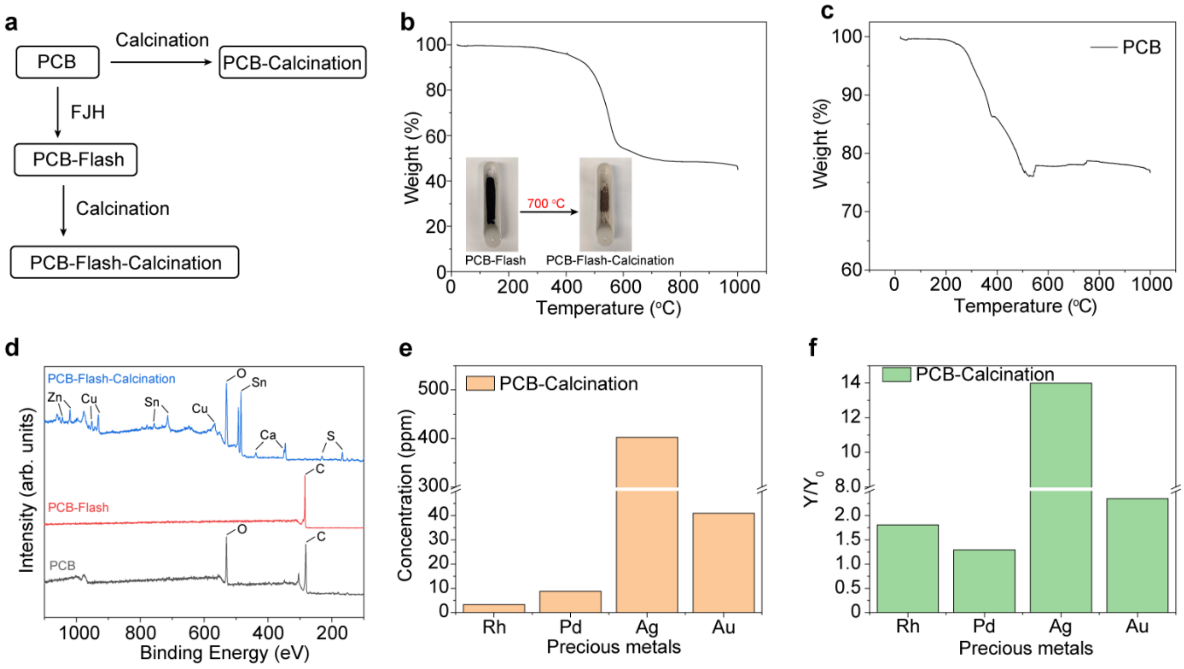

Supplementary Fig. 17 | Recovery of precious metal by flash Joule heating (FJH) and calcination. (a) Different processes for the recovery of precious metals from printed circuit board (PCB). (b) Thermogravimetric analysis (TGA) curve of PCB-Flash in air. Inset, the picture of PCB-Flash, and PCB-Flash-Calcination. The TGA curve shows that the PCB-Flash started to lose weight at ~400 °C and remains stable at ~800 °C. (c) TGA curve of PCB. (d) X-ray photoemission spectroscopy (XPS) of PCB, PCB-Flash, and PCB-Flash-Calcination. The XPS of PCB shows mostly C and some inorganic signals. The XPS of PCB-Flash shows mostly C signals, indicating that O was removed by the FJH process, and the inorganic element peaks are not detected, presumably because the inorganics were covered by carbon during the FJH process. The XPS of PCB-Flash-Calcination show abundant elemental signals, demonstrating the removal and exposure of inorganic materials. (e) Concentration of precious metals in PCB-Calcination. (f) Improvement of leaching yield by calcination. Y_0 and Y mean the recovery yield by leaching PCB and PCB-Calcination, respectively.

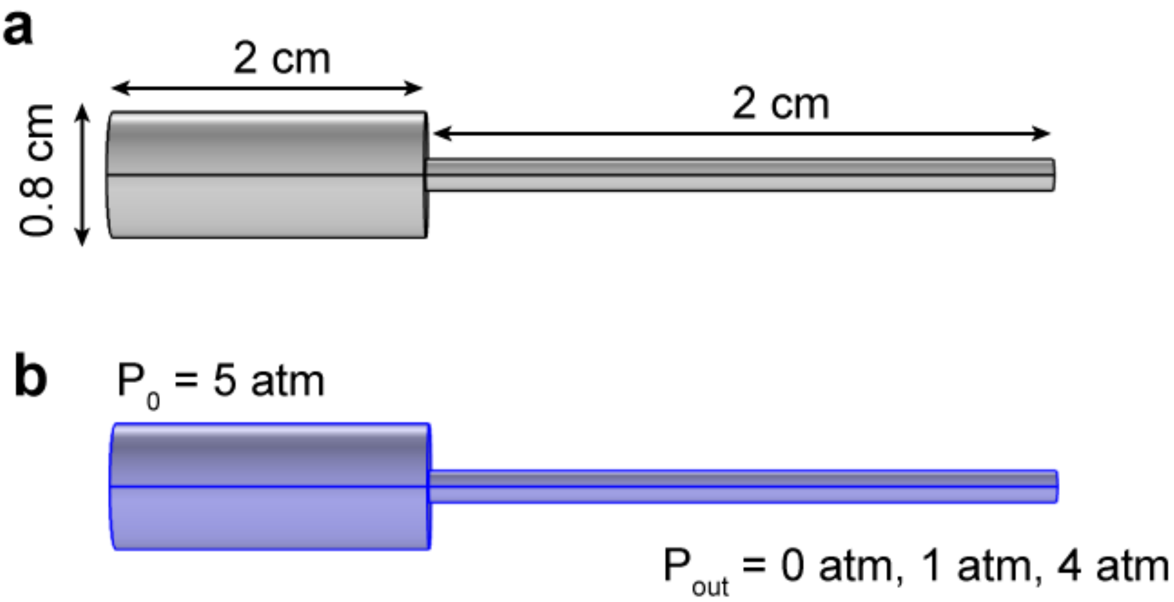

Supplementary Fig. 18 | Gas flow simulation. (a) Geometrical conditions. (b) Boundary conditions.

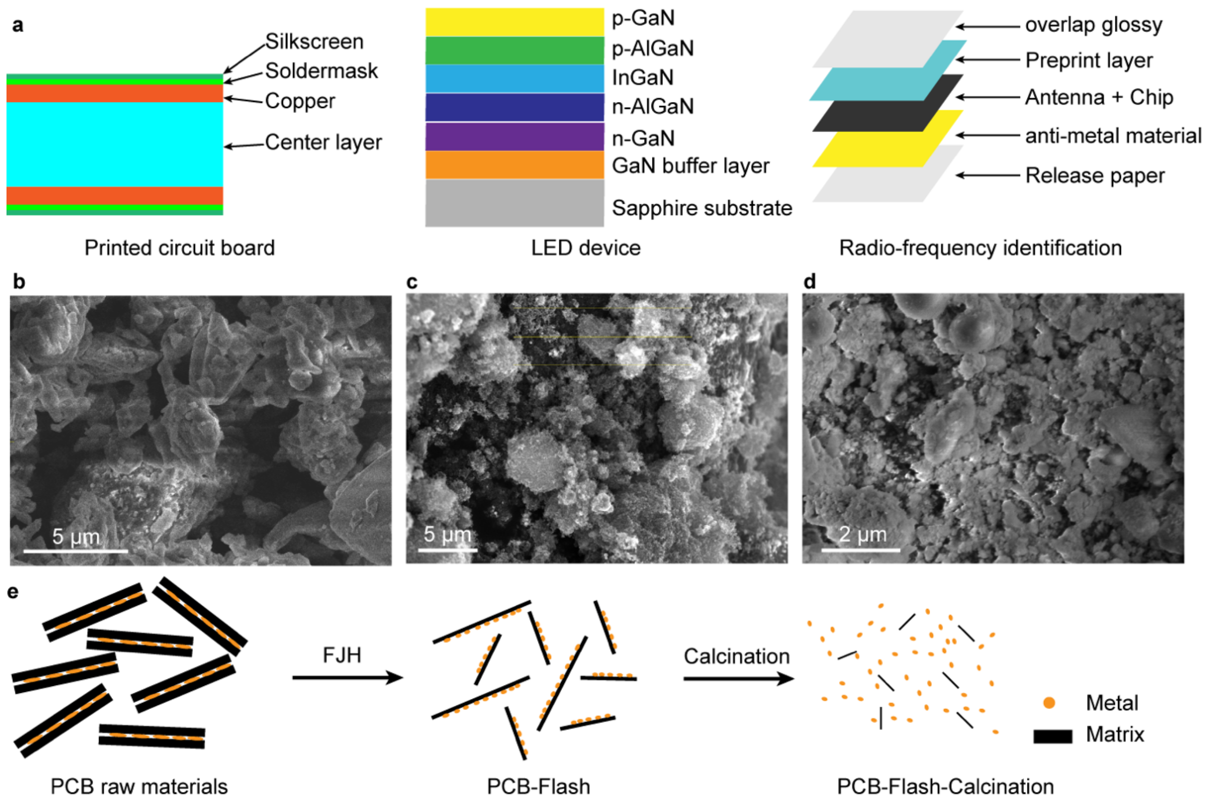

Supplementary Fig. 19 | Mechanism of the improvement of leaching efficiency by flash Joule heating (FJH). (a) Scheme of the laminated configuration of several types of electronics. (b) Scanning electron microscopy (SEM) image of printed circuit board (PCB) powders. (c) SEM image of PCB-Flash. (d) SEM image of PCB-Flash-Calcination. (e) The scheme of morphological and structure changes of PCB during the FJH and calcination process.

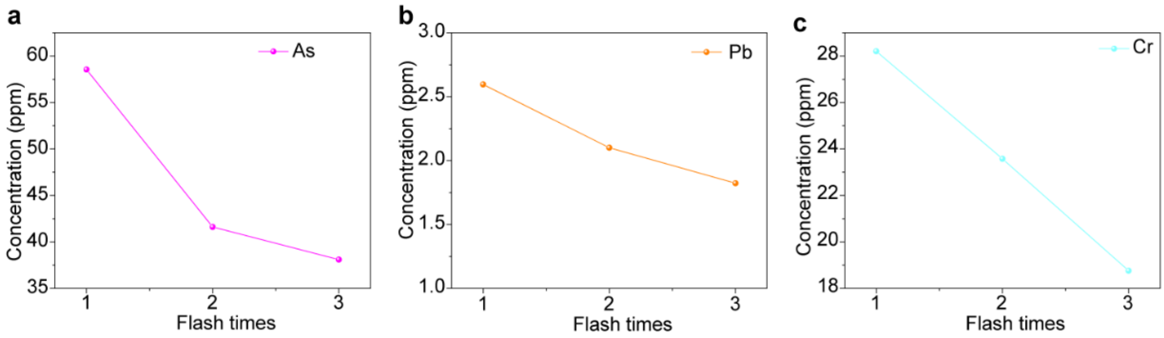

Supplementary Fig. 20 | Concentration of remaining heavy metals after multiple flash Joule heating (FJH) reactions. (a) Concentration of As. (b) Concentration of Pb. (c) Concentration of Cr. Each FJH is 1 s.

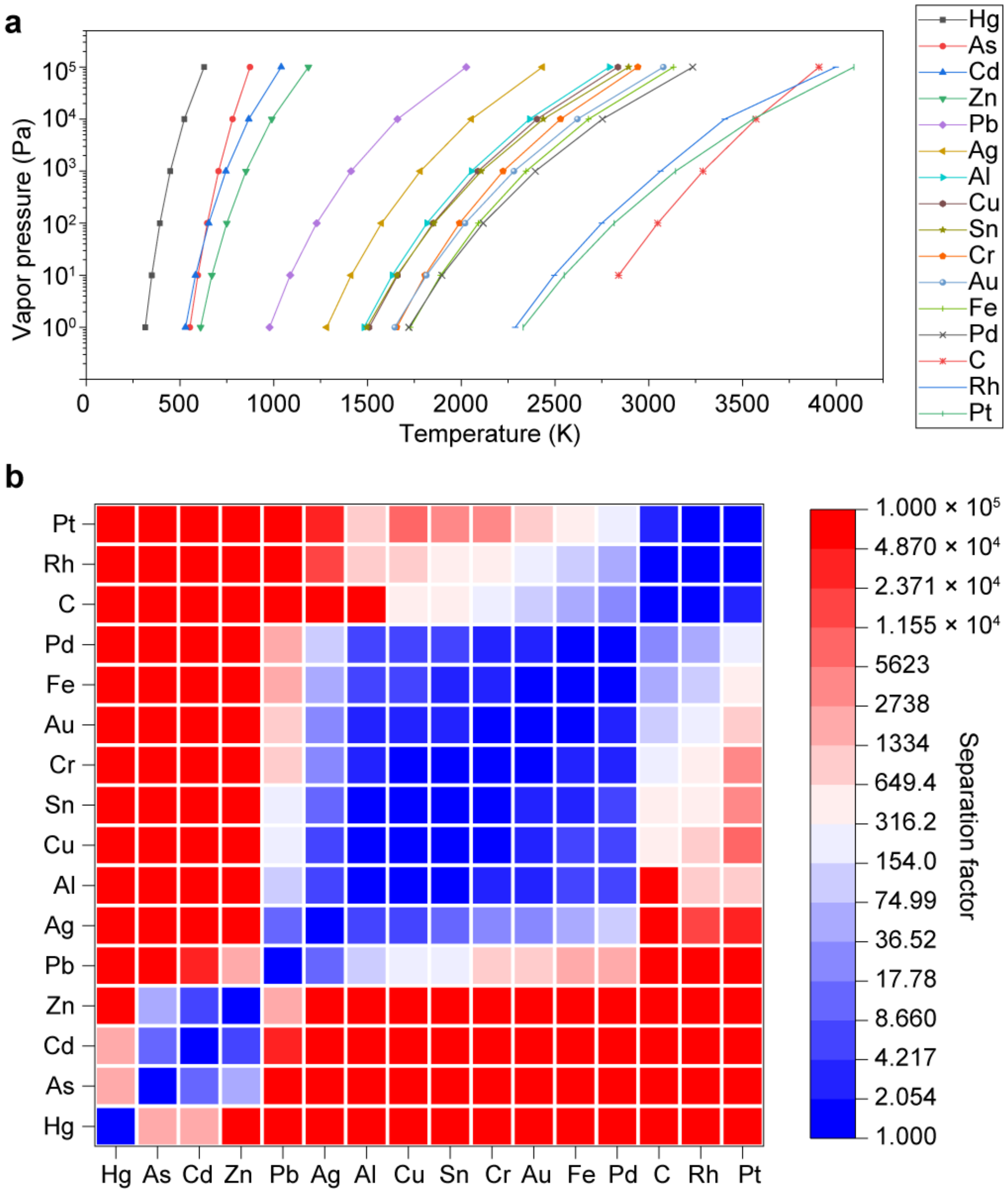

Supplementary Fig. 21. Theoretical separation factor of the evaporative separation process. (a) Vapor pressure-temperature relationship of 16 representative elements. (b) Heatmap of the theoretical separation factors for the 16 elements.

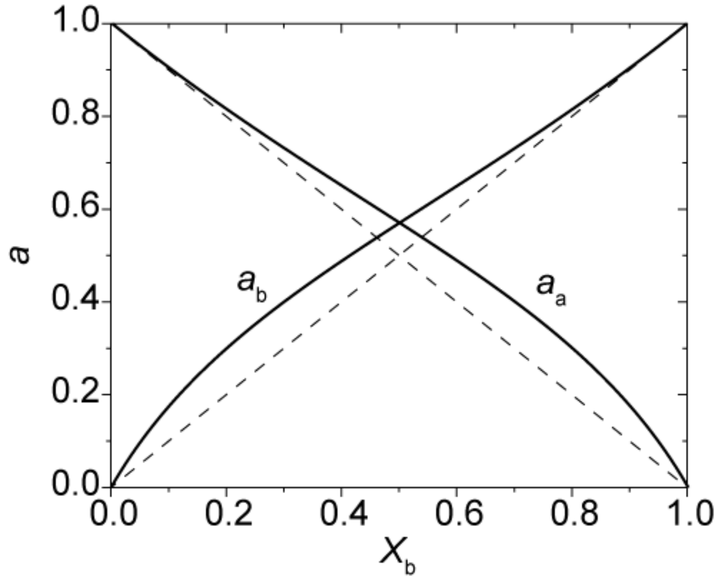

Supplementary Fig. 22. Activity-composition relationship.

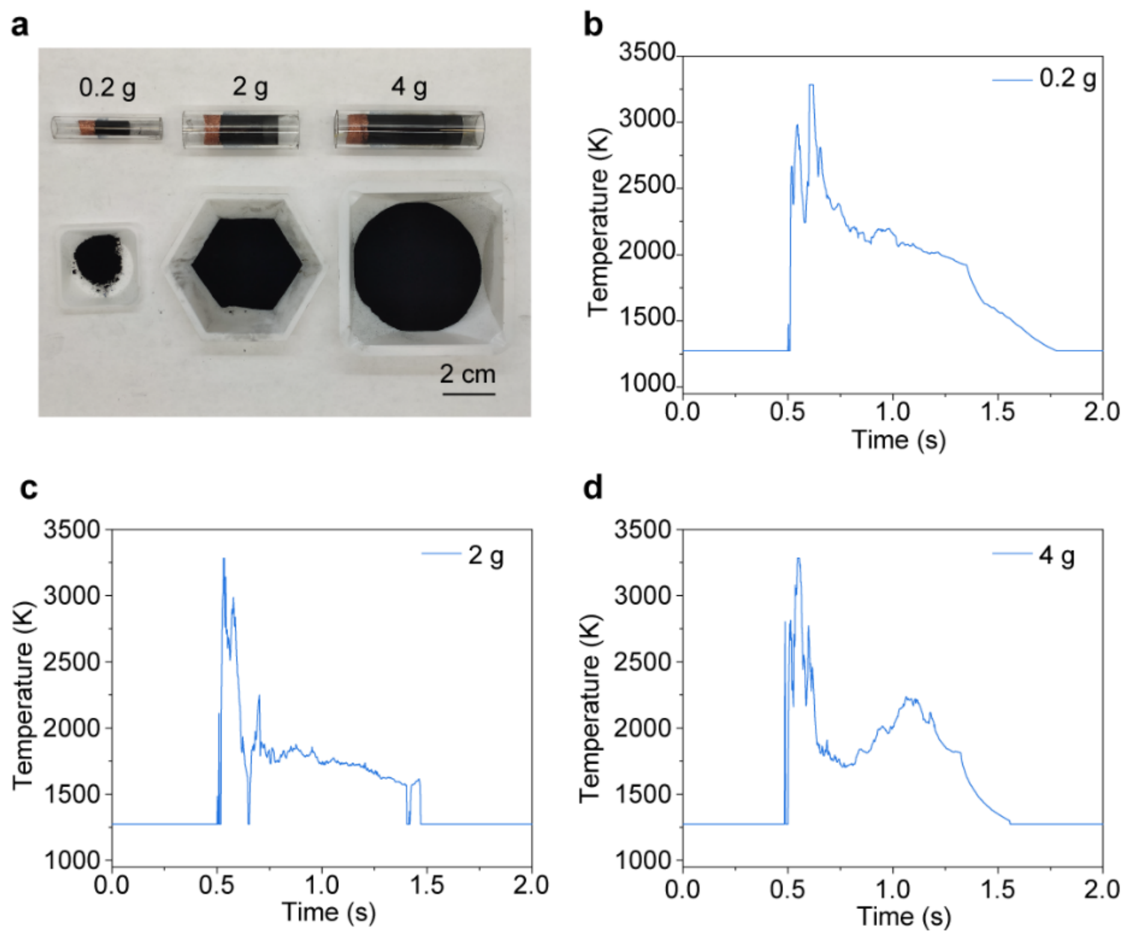

Supplementary Fig. 23. Scaling up of the flash Joule heating (FJH) process. (a) Picture of the samples treated with the condition of m_0 = 0.2 g, V_0 = 150 V, and C_0 = 0.06 F (left), m_1 = 2 g, V_1 = 150 V, and C_1 = 0.6 F (middle), m_2 = 4 g, V_2 = 300 V, and C_2 = 0.6 F (right). (b-d) Realtime temperature curves for samples with mass of m_0 = 0.2 g (b), m_1 = 2 g (c), and m_2 = 4 g (d).

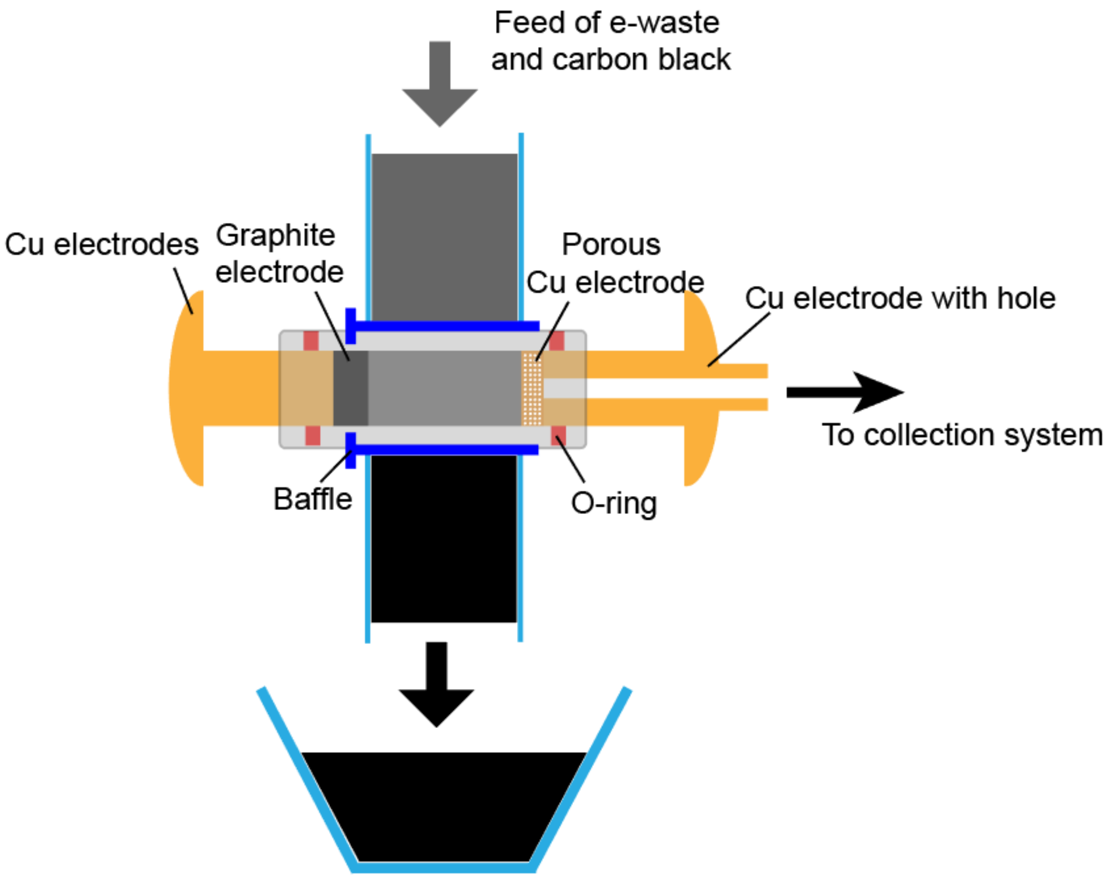

Supplementary Fig. 24. The scheme of a continuous flash Joule heating (FJH) reactor for e-waste processing.

Supplementary Table 1. Parameters for FJH under vacuum.

Precursors	Mass Ratio	Mass (mg)	Resistance (Ω)	Voltage (V)	Time (s)
PCB:CB, 1#	2:1	300	1.8	150	1
PCB:CB, 2#	2:1	300	2.0	150	1
PCB:CB, 3#	2:1	300	2.5	150	1
PCB:CB:NaCl, 1#	1:2:3	300	2.3	150	1
PCB:CB:NaCl, 2#	1:2:3	300	1.9	150	1
PCB:CB:NaCl, 3#	1:2:3	300	1.8	150	1
PCB:CB:KCl, 1#	1:2:3	300	22	150	1
PCB:CB:KCl, 2#	1:2:3	300	18	150	1
PCB:CB:KCl, 3#	1:2:3	300	14	150	1
PCB:CB:PVC, 1#	1:2:3	300	2.0	150	1
PCB:CB:PVC, 2#	1:2:3	300	2.5	150	1
PCB:CB:PVC, 3#	1:2:3	300	2.5	150	1
PCB:CB:CPVC, 1#	1:2:3	200	3.0	150	1
PCB:CB:CPVC, 2#	1:2:3	200	3.0	150	1
PCB:CB:CPVC, 3#	1:2:3	200	3.2	150	1
PCB:CB:NaF, 1#	1:2:3	200	1.5	150	1
PCB:CB:NaF, 2#	1:2:3	200	1.0	150	1
PCB:CB:NaF, 3#	1:2:3	200	1.0	150	1
PCB:CB:PTFE, 1#	1:2:3	200	2.0	150	1
PCB:CB:PTFE, 2#	1:2:3	200	2.2	150	1
PCB:CB:PTFE, 3#	1:2:3	200	2.2	150	1
PCB:CB:NaI, 1#	1:2:3	200	0.6	150	1
PCB:CB:NaI, 2#	1:2:3	200	0.6	150	1
PCB:CB:NaI, 3#	1:2:3	200	0.5	150	1
PCB:CB:NaF:NaCl:NaI, 1#	1:2:1:1:1	200	0.2	150	1
PCB:CB:NaF:NaCl:NaI, 2#	1:2:1:1:1	200	1	150	1
PCB:CB:NaF:NaCl:NaI, 3#	1:2:1:1:1	200	0.5	150	1

Supplementary Table 2. Parameters for FJH under pressure.

Precursors	Mass Ratio	Mass (mg)	Resistance (Ω)	Pressure	Voltage (V)	Time (s)	Mass after FJH (mg)
PCB:CB	2:1	200	1.0	1 bar	10	1	196
PCB:CB	2:1	200	1.3	1 bar	30	1	196
PCB:CB	2:1	200	1.0	1 bar	50	1	180
PCB:CB	2:1	200	1.0	1 bar	100	1	158
PCB:CB	2:1	200	1.0	1 bar	120	1	115
PCB:CB	2:1	200	1.0	vacuum	120	1	65
PCB:CB	2:1	200	1.0	1 bar	120	1	115
PCB:CB	2:1	200	1.0	2 bar	120	1	142
PCB:CB	2:1	200	1.0	3 bar	120	1	155
PCB:CB	2:1	200	1.0	4 bar	120	1	165

Supplementary Table 3. The calculated separator factor of the evaporative separation process.

	Hg	As	Cd	Zn	Pb	Ag	Al	Cu	Sn	Cr	Au	Fe	Pd	C	Rh	Pt
Hg	1	2309	2309	50000	100000	100000	100000	100000	100000	100000	100000	100000	100000	100000	100000	100000
As	2309	1	9.13	69.9	100000	100000	100000	100000	100000	100000	100000	100000	100000	100000	100000	100000
Cd	2309	9.13	1	5.71	28089	100000	100000	100000	100000	100000	100000	100000	100000	100000	100000	100000
Zn	50000	69.9	5.71	1	2066	100000	100000	100000	100000	100000	100000	100000	100000	100000	100000	100000
Pb	100000	100000	28089	2066	1	12.1	129	175	206	680	913	1961	2439	100000	100000	100000
Ag	100000	100000	100000	100000	12.1	1	6.69	8.64	10	19.8	35	56.1	78.7	100000	19083	34130
Al	100000	100000	100000	100000	129	6.69	1	1.25	1.65	2.31	4.21	5.5	8.32	100000	735	1235
Cu	100000	100000	100000	100000	175	8.64	1.25	1	1.3	1.8	3.35	4.43	6.5	513	870	10000
Sn	100000	100000	100000	100000	206	10	1.65	1.3	1	1.3	2.45	3.39	5.19	349	584	5618
Cr	100000	100000	100000	100000	680	19.8	2.31	1.8	1.3	1	1.96	2.57	3.89	247	401	3141
Au	100000	100000	100000	100000	913	35	4.21	3.35	2.45	1.96	1	1.28	2.16	90.6	160	754
Fe	100000	100000	100000	100000	1961	56.1	5.5	4.43	3.39	2.57	1.28	1	1.59	61.1	105	443
Pd	100000	100000	100000	100000	2439	78.7	8.32	6.5	5.19	3.89	2.16	1.59	1	31.7	60.2	172
C	100000	100000	100000	100000	100000	100000	100000	513	349	247	90.6	61.1	31.7	1	1.4	2.18
Rh	100000	100000	100000	100000	100000	19083	735	870	584	401	160	105	60.2	1.4	1	1.48
Pt	100000	100000	100000	100000	100000	34130	1235	10000	5618	3141	754	443	172	2.18	1.48	1

Note: The theoretical separation factors are calculated based on the vapor pressure difference of pure metals. They represent practical values for trace metals separation from abundant metals. For the separation of abundant metals, the values should be corrected according to their activity in the alloy melt.

Supplementary Table 4. The separation factors of precious metals.

	Rh	Pd	Ag	Au
Rh	1	1.29	9.5	3.1
Pd	1.29	1	12.3	2.4
Ag	9.5	12.3	1	29.2
Au	3.1	2.4	29.2	1

Supplementary Table 5. The separation factors of precious metals by using NaCl additives

	Rh	Pd	Ag	Au
Rh	1	1.44	3.0	840
Pd	1.44	1	4.33	58.3
Ag	3.0	4.33	1	253
Au	840	58.3	253	1

Supplementary Table 6. The separation factors of precious metals by using NaF additives

	Rh	Pd	Ag	Au
Rh	1	1.52	1.8	146
Pd	1.52	1	1.19	96
Ag	1.8	1.19	1	81
Au	146	96	81	1

Supplementary Table 7. The separation factors of precious metals by using NaI additives

	Rh	Pd	Ag	Au
Rh	1	1.05	1.08	1.59
Pd	1.05	1	1.03	1.51
Ag	1.08	1.03	1	1.48
Au	1.59	1.51	1.48	1

Supplementary Table 8. Standards for ICP-MS.

**Standards**	**Elements**	**Concentrations**	**Matrix**
Periodic table mix 1	Al, As, Ba, Be, Bi, B, Ca, Cd, Cs, Cr, Co, Cu, Ga, In, Fe, Pb, Li, Mg, Mn, Ni, P, K, Rb, Se, Si, Ag, Na, Sr, S, Te, Tl, V, and Zn	10 mg/L	10% HNO_3
Periodic table mix 2	Au, Ge, Hf, Ir, Mo, Nb, Pd, Pt, Re, Rh, Ru, Sb, Sn, Ta, Ti, W, and Zr	10 mg/L	5% HCl, 1% HF
Periodic table mix 3	Sc, Y, La, Ce, Pr, Nd, Sm, Eu, Gd, Tb, Dy, Ho, Er, Tm, Yb and Lu	10 mg/L	5% HNO_3
Mercury	Hg	1000 mg/L	12% HNO_3

Supplementary References

1 Bernardis, F. L., Grant, R. A. & Sherrington, D. C. A review of methods of separation of the platinum-group metals through their chloro-complexes. *React. Funct. Polym.* **65**, 205-217 (2005).

2 Ueda, T., Ichiishi, S., Okuda, A. & Matsutani, K. in *Metal Sustainability: Global Challenges, Consequences, and Prospects* (ed Reed M. Izatt) 333-360 (John Wiley & Sons, Ltd, 2016).

3 Yousif, A. M. Recovery and Then Individual Separation of Platinum, Palladium, and Rhodium from Spent Car Catalytic Converters Using Hydrometallurgical Technique followed by Successive Precipitation Methods. *J. Chem.* **2019** (2019).

4 Charlesworth, P. Separating the platinum group metals by liquid-liquid extraction. *Platinum Met. Rev.* **25**, 106-112 (1981).

5 Metal Separation Technologies beyond 2000: Integrating Novel Chemistry with Processing, Symposium Proceeding, Hawaii, June 13-18, TMS, 1999, pp. 357-370.

6 Dong, Z. H. *et al.* Vaporization of Ni, Al and Cr in Ni-Base Alloys and Its Influence on Surface Defect Formation During Manufacturing of Single-Crystal Components. *Metall Mater. Trans. A* **51**, 309-322 (2020).

7 Balaji, R. & Senophiyah-Mary, J. in *Urban Mining and Sustainable Waste Management* (ed Sadhan Kumar Ghosh) 59-67 (Springer Singapore, 2020).

8 Theo, L. in *Proceedings of the 1998 IEEE International Symposium on Electronics and the Environment. ISEE - 1998 (Cat. No.98CH36145).* 42-47.

9 Luong, D. X. *et al.* Gram-scale bottom-up flash graphene synthesis. *Nature* **577**, 647-651 (2020).

10 Stanford, M. G. *et al.* Flash Graphene Morphologies. *ACS Nano* **14**, 13691-13699 (2020).

11 Algozeeb, W. A. *et al.* Flash graphene from plastic waste. *ACS Nano* **14**, 15595-15604 (2020).

12 Okamoto, H., Schlesinger, M. E., Mueller, E. M., ASM Handbook Volume 3: Alloy Phase Diagrams, (ASM International, Ohio, 2016)

13 *CRC Handbook of Chemistry and Physics*. Internet Version 2005 edn, (CRC Press, 2005).

14 Jayne, D. T., Fatemi, N. S. & Weizer, V. G. An X-Ray Photoelectron-Spectroscopy Study of Auxiny Alloys. *J. Vac. Sci. Technol. A* **9**, 1410-1415 (1991).

15 Andersson, S. L. T., Watters, K. L. & Howe, R. F. An XPS Study of $Rh_6(Co)_{16}$-Al_2O_3. *J. Catal.* **69**, 212-215 (1981).

16 Sleigh, C., Pijpers, A. P., Jaspers, A., Coussens, B. & Meier, R. J. On the determination of atomic charge via ESCA including application to organometallics. *J. Electron Spectrosc.* **77**, 41-57 (1996).
